# Charge Dynamics of a CuO Thin Film on Picosecond to Microsecond Timescales Revealed by Transient Absorption Spectroscopy

**DOI:** 10.1021/acsami.2c22595

**Published:** 2023-03-30

**Authors:** Mona Asadinamin, Aleksandar Živkovic, Susanne Ullrich, Henning Meyer, Yiping Zhao

**Affiliations:** † Department of Physics and Astronomy, University of Georgia, Athens, Georgia 30602, United States; ‡ Department of Earth Sciences, Utrecht University, Princetonlaan 8a, 3548 CB Utrecht, The Netherlands

**Keywords:** CuO, transient absorption spectroscopy (TAS), model, energy diagram, rate equations, photocatalysts

## Abstract

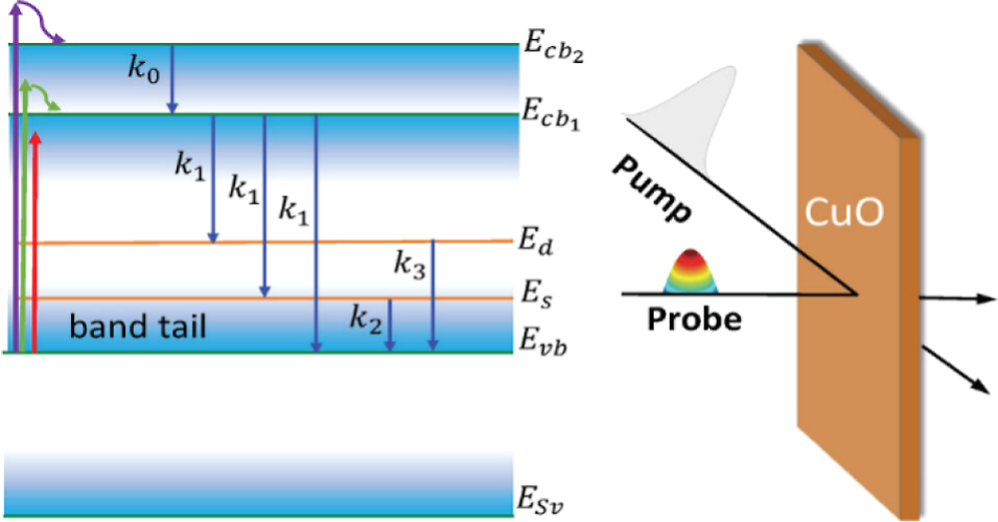

Understanding the mechanism of charge dynamics in photocatalysts is the key to design and optimize more efficient materials for renewable energy applications. In this study, the charge dynamics of a CuO thin film are unraveled via transient absorption spectroscopy (TAS) on the picosecond to microsecond timescale for three different excitation energies, i.e., above, near, and below the band gap, to explore the role of incoherent broadband light sources. The shape of the ps-TAS spectra changes with the delay time, while that of the ns-TAS spectra is invariant for all the excitation energies. Regardless of the excitations, three time constants, τ_1_ ∼ 0.34–0.59 ps, τ_2_ ∼ 162–175 ns, and τ_3_ ∼ 2.5–3.3 μs, are resolved, indicating the dominating charge dynamics at very different timescales. Based on these observations, the UV–vis absorption spectrum, and previous findings in the literature, a compelling transition energy diagram is proposed. Two conduction bands and two defect (deep and shallow) states dominate the initial photo-induced electron transitions, and a sub-valence band energy state is involved in the subsequent transient absorption. By solving the rate equations for the pump-induced population dynamics and implementing the assumed Lorentzian absorption spectral shape between two energy states, the TAS spectra are modeled which capture the main spectral and time-dependent features for *t* > 1 ps. By further considering the contributions from free-electron absorption during very early delay times, the modeled spectra reproduce the experimental spectra very well over the entire time range and under different excitation conditions.

## 1 Introduction

Photocatalysts have various important energy and environmental applications, such as solar-water splitting, solar CO_2_ conversion, and antimicrobial/antiviral activity.^1,2^ All these applications involve at least two processes: photocarrier generation in which light excites the photocatalyst to generate radicals such as reactive oxygen species (ROS) and photocatalysis reactions in which ROS react with the desired chemical species surrounding the photocatalyst.^3,4^ In the first process, the dynamics of the photoexcited charge carriers in photocatalysts is the limiting factor which determines the concentration of the ROS products, while in the second process the reaction kinetics and dynamics play the most important role. Intrinsically, the charge dynamics occur on different timescales; i.e., the relaxation of hot charge carriers occurs in less than 1 picosecond (ps), recombination and de-trapping occur in a nanosecond (ns) to microsecond (μs) or even millisecond (ms) timescale, while photocatalysis reactions, i.e., reduction, oxidation, or redox reactions, occur in μs to s timescale.^5−7^ Clearly, there is a mismatch between the timescale of the intrinsic dynamics of photocarriers and chemical reactions, which demands a thorough investigation of their relationship across a broad timescale. Therefore, understanding the dynamics of photoexcited charge carriers over an extended time range is critical. One of the most frequently used tools to investigate photocarrier dynamics is transient absorption spectroscopy (TAS), which provides essential information about both the time-dependent and spectral characteristics of the charge dynamics.

So far, most TAS studies are mainly focused on either the ultra-fast (fs to ps) or long (ns to s) timescales which can only provide an incomplete picture of the charge dynamics.^8,9^ In addition, many of them are only concentrated on the time-dependent behavior at specific probe photon energies (i.e., selected wavelengths in the TAS spectrum) and neglect the overall spectral changes as a function of time.^7,10^ In principle, the time-dependent behavior of the spectra in different wavelength regions should be different because they may correspond to different transitions and reveal important information on the energy states involved within the photocatalysts. However, only a handful of papers reported a simultaneous analysis of spectral and time-dependent behavior. For example, Cooper et al. emphasized the importance of simultaneous disentangling of spectral and kinetics behavior in TAS spectra and proposed a comprehensive energy diagram for BiVO_4_ by combining optical properties of the material with the known mechanisms of band edge broadening, band-gap alterations, and free-carrier absorption.^11^ Yoshihara et al. analyzed the spectral behavior of TiO_2_ films to extract the contributions of electrons and holes in the charge dynamics.^12^ Kollenz et al. introduced a deep-learning-based approach to elucidate the charge dynamics of materials by simultaneous analysis of spectral and kinetic dependence of TAS spectra of lycopene in tetrahydrofuran.^13^ Furthermore, in many photocatalytic-based applications, an incoherent broadband light source is used, enabling the photocatalyst to utilize different electronic transitions triggered by different energies. Thus, to better understand the roles of different energy states in photocatalysis processes under a broadband illumination, it is beneficial to investigate the photocarrier charge dynamics at different excitation energies. Therefore, in order to obtain a complete picture of the charge dynamics of a photocatalyst, one should conduct a broad timescale investigation, from ps to μs (or even ms); perform simultaneous analysis of the spectral and time-dependent behaviors under different excitation energies; and explain the observed results comprehensively.

Here, we attempt to implement the above-mentioned strategy to investigate the charge dynamics of a CuO thin film using ps- and ns-TAS. CuO is an earth-abundant, photostable, sustainable p-type metal oxide^14−17^ and has an optical band gap (*E*_g_) of 1.5–2.2 eV.^18−21^ It has been widely used for photocatalytic applications^22−25^ and photoelectrochemical cells^26,27^ and has been reported as an excellent candidate for large-scale photovoltaic deployment.^28^ However, there have been only a few experimental studies on the charge dynamics of CuO. Othonos et al. studied the charge carrier relaxation in CuO nanowires (*E*_g_ = 1.5 eV) via picosecond—transient absorption spectroscopy (ps-TAS) (only up to 8 ps) using an excitation energy *h*ν_p_ (*h* is the Planck’s constant and ν_p_ is the frequency of the excitation beam) of 3.1 eV.^29^ By analyzing the time-dependent behavior at different probe energies, they reported two time constants, 0.4, and 2.1 ps, which were associated with the hole relaxation from the sub-valence band (SVB) states to the hole states above the valence band (VB).^29^ Born et al. studied the ultra-fast charge dynamics of CuO nanocrystals (*E*_g_ = 1.55 eV) (only up to 20 ps) with *h*ν_p_ = 1.6 eV, i.e., exciting electrons near the conduction band (CB) edge. The authors only probed the charge dynamics at 0.8 eV, corresponding to a mid-gap energy, which was claimed to be sensitive to the carrier dynamics of the holes near the VB. Based on their time-dependent analysis, three time constants were reported: 330–640 fs corresponding to the momentum relaxation via carrier–carrier scattering in the VB, 2 ps for the energy relaxation via carrier-phonon scattering within the VB, and 50 ps due to the trapping and recombination. Shenje et al. investigated the photocarrier dynamics of a CuO thin film (*E*_g_ = 2.2 eV) up to 150 ps with *h*ν_p_ = 3.1 eV.^19^ Three time constants were obtained, 250 fs, 2.5 ps, and >150 ps, which were attributed to the previously suggested mechanisms by Born et al.^18^ The authors also proposed a possible energy diagram and electronic transitions for the CuO thin film by considering the Urbach tail which represented defect states at 0.2 eV above the VB. Although these previous studies have revealed some essential features of the charge dynamics of CuO, there is a discrepancy in the reported time constants, which could be due to the variations in materials, the limited timescale of the measurements, and the different excitation energies employed by these groups. Thus far, no comprehensive study has been carried out to understand the overall time-dependent spectral behavior in CuO TAS nor extended to a broad timescale, i.e., ps to μs timescale. Moreover, all the previous studies are limited to a single excitation energy. Clearly, a comprehensive understanding and a solid picture of the carrier dynamics of CuO are yet to be determined.

Herein, we carried out systematic experiments to probe the charge dynamics of a CuO thin film from the ps to μs timescale using both ps-TAS and ns-TAS under three different excitation energies: above, near, and below *E*_g_. The overall TAS spectra revealed three time constants, ∼0.5 ps, ∼170 ns, and ∼2.8 μs, which dictated three time evolution regions: ultra-fast, quasi-static, and recovery stages. The TAS spectral features indicated that there exist two intrinsic absorptions near and above *E*_g_, one at ∼2.2 eV (*E*_g_) and the other at ∼2.7 eV. Based on these experimental results and density functional theory calculations in the literature, a comprehensive transition energy diagram with the energy states contributing to the TAS experiments and possible electronic transitions were proposed. Rate equations based on the above assumptions were established, and the time-dependent spectra, which were based on Lorentzian spectral functions for each allowed electron transition, were found to reasonably agree with the experimental findings. These results support our proposed strategy; i.e., to gain a complete picture of the charge dynamics of a photocatalyst, one needs to conduct systematic TAS investigations over an extended time range with varied excitation energies and thoroughly analyze the time-dependent spectra. Such a strategy is also expected to help us establish better insights into the carrier dynamics of a photocatalyst and the corresponding photocatalytic reactions.

## 2 Experimental Section

### 2.1 Materials

Copper shots (99.9+%) were purchased from Kurt J. Lesker (Clairton, PA). Cleaned glass microscope slides (Gold Seal catalog no. 3010) were used as the substrates for material deposition. Deionized water (18 MΩ cm) was used to prepare all solutions.

### 2.2 Sample Preparation

A 50 nm Cu thin film was deposited onto the glass substrates by a custom-designed electron-beam deposition system. Glass substrates (3.3 × 1 cm^2^) were cleaned by a piranha solution using a 4:1 mixture of sulfuric acid (H_2_SO_4_) and hydrogen peroxide (H_2_O_2_) solutions, boiled for about 10 min, rinsed 6 times by deionized water, dried under nitrogen flow, and subsequently mounted into the deposition chamber. The cleaned glass substrates were loaded onto the deposition chamber, and the chamber was pumped down to a base pressure of 3 × 10^–6^Torr. During the deposition, the pressure was maintained at about ≤6 × 10^–6^ Torr. The deposition rate (0.02 nm s^–1^) and thickness of the Cu thin film (50 nm) were monitored by a quartz crystal microbalance. The as-deposited Cu thin films were then oxidized in a quartz tube furnace (Lindberg/Blue M Company) at a preset temperature of 380 °C, under an oxygen flow (20 SCCM) for 3 h. During the annealing, the temperature was ramped at a rate of 5 °C min^–1^. After oxidation, the resulting CuO thin film was determined to be 100 nm thick by atomic force microscopy (Park Systems NX-10 AFM). Three other sets of samples were prepared over a year to confirm reproducibility.

### 2.3 General Characterization

The crystal structure of the oxidized sample was characterized using a PANalytical X’Pert- PRO MRD X-ray diffractometer (XRD) at a fixed incidence angle of 0.5°. The XRD pattern was recorded with a Cu *K*α_1_ radiation (λ = 0.154 nm) in the 2θ range from 20 to 80° in increments of 0.010°. The optical transmission spectra of the sample were measured using a dual-beam UV–visible (UV–vis) spectrophotometer (Shimadzu- UV2450) over the energy range of 1.5–4.1 eV (300–820 nm wavelength range).

### 2.4 Picosecond-Transient Absorption Spectroscopy

A custom-built setup was used for the ps-TAS measurements. The detail of the setup is explained elsewhere.^30^ Briefly, an 800 nm laser pulse with a temporal width of 130 fs was initially generated by a commercially available regenerative amplifier (Coherent Inc Legend Elite seeded by a Mira Optima 900) with a repetition frequency of 1 kHz; the output was used to feed a traveling wave optical parametric amplifier (TOPAS-C) in order to generate the excitation beam at *h*ν_p_ = 3.5 eV (355 nm), 2.2 eV (565 nm), and 1.7 eV (740 nm) with pulse energies of 2, 5.7, and 4.3 μJ, respectively.

At a specific probe beam frequency ν and a time delay *t*, the absorption difference spectrum, i.e., the TAS spectrum Δ*A*(ν,*t*), was determined by
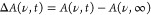
1where *A*(ν,*t*) and *A*(ν,∞) are the absorption spectra at a time delay *t* and in a steady state, respectively. These absorption spectra were determined by taking the negative natural logarithm of the measured transmission spectra with and without the excitation beam. The Surface Xplorer (Ultrafast Systems LLC) software package was used to process the raw data.

### 2.5 Nanosecond-Transient Absorption Spectroscopy

A transient absorption spectrometer for the ns to μs timescale was set up using the output of a Nd-YAG (Spectra Physics GCR150-10) pumped dye laser (LAS LDL2005) as the excitation source. The system was operated at 10 Hz and provided excitation pulses of about 5 ns duration. In case the output of the dye laser was frequency doubled in a non-linear crystal (KDP or BBO), the fundamental was separated from the second harmonic using a CaF_2_ Pellin–Broca prism. In this setup, the accessible excitation wavelengths ranged from 450 to 740 nm and from 225 to 370 nm. A continuous IR filtered output of a Xe arc lamp (Oriel 66001) was used as the probe light. For ns-TAS measurements, the pump beam hit the sample under an angle of 6° from the normal, while the probe light propagated along the normal. The divergent probe beam was collimated before and after the sample with 200 and 300 mm plano-convex lenses, respectively. The probe beam after the sample was analyzed with a monochromator (ARC SpectraPro 150) and detected with a photomultiplier (Electron Tubes: 9813B; housing B2F/RFI). In order to reduce the load on the photomultiplier tube (PMT), a phase-locked chopper (Thorlabs MC 2000B-EC) with a 10% duty cycle blade was used to modulate the probe beam. The time-dependent signal of the PMT was recorded and averaged with a fast digital scope (Tektronix DS684C). The complete system had an overall time response of 15 ns. A computer-controlled shutter allowed to simultaneously block the pump and probe beams, thus realizing a baseline subtraction that efficiently removed the electrical noise due to the firing of the YAG laser. Background signals due to scattered light from the pump laser were suppressed using different combinations of dichroic filters.

## 3 Results and Discussion

### 3.1 Structure and Optical Properties of the CuO Thin Film

**Figure 1:**
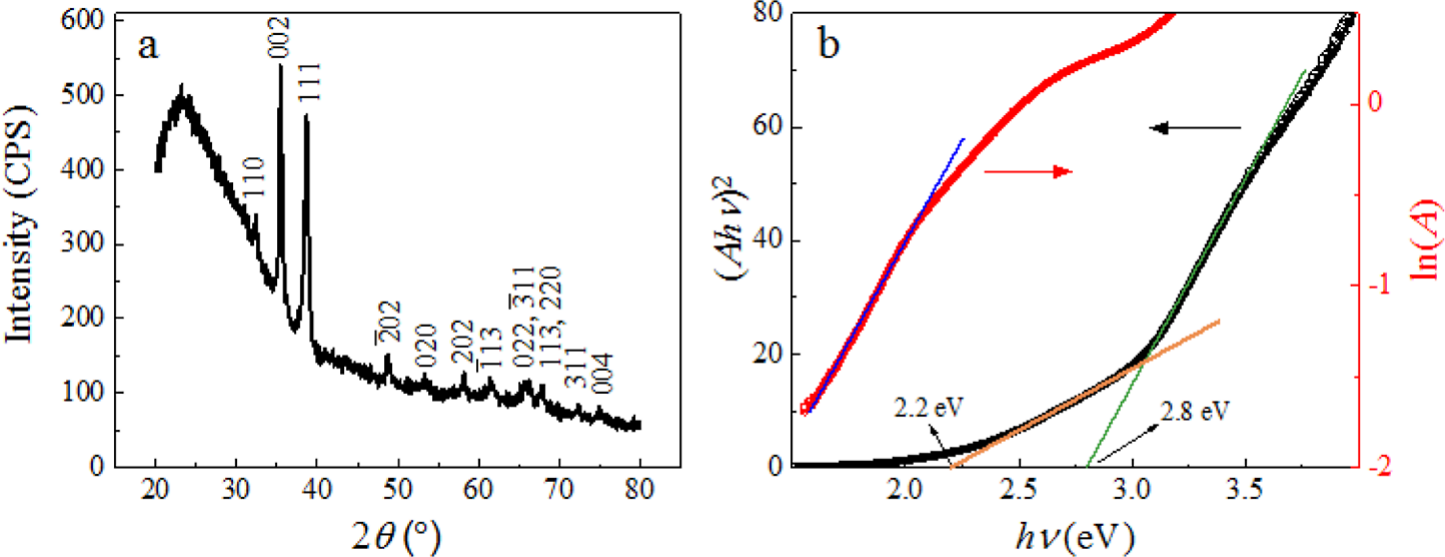
(a) XRD pattern and (b) optical analysis of the CuO thin film. The black curve (left axis) in (b) corresponds to the Tauc plot, and the red curve (right axis) is a semi-log plot of absorption *A*.

Figure 1a shows the XRD pattern of the CuO thin film. All of the observed peaks correspond to the characteristic crystalline planes of CuO (PDF#80-1268), and the thin film does not contain any other phases of copper oxide compounds within the detection limit of the XRD system. Scherrer’s equation was used to estimate the crystallite size *D*,^31^
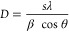
2where *s* is the shape factor, λ is the X-ray wavelength, β is the full-width-at-half-maximum of the dominant peaks, and θ is the angle of the dominant peak. λ = 0.154 nm, and for the CuO thin film, the shape factor is assumed to be *s* = 0.9.^32^ The dominant peaks in Figure 1a at (002) and (111) surfaces are at θ = 17.7 and 19.3°, where β = 0.39 and 0.50°, respectively. The estimated crystallite size is *D* = 16–18 nm, within the range of the reported values for CuO thin films in the literature.^33^ The relatively small average crystallite size compared to the thickness of the CuO film (100 nm) indicates the existence of many interfacial boundaries in the thin film, which breaks the periodicity of the crystal and induces more defect, surface, and amorphous states.

The typical optical absorption spectrum of the CuO thin film is shown in Figure S1. It exhibits a non-zero absorption at photons energies *h*ν as small as 1.5 eV. The absorption monotonically increases from 1.5 to 3 eV and then increases more rapidly for *h*ν > 3 eV. The direct band gap, *E*_g_, of the film is estimated by a Tauc plot shown in Figure 1b (the black curve) according to
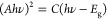
3where *A* is the absorption and *C* is the proportionality constant. The black curve in Figure 1b is the (*Ah*ν)^2^*versush*ν plot and demonstrates two linear regions: the one fitted by an orange line gives a band gap of *E*_g_ = 2.2 ± 0.08 eV, whereas the other region fitted by the green line results in a band-gap-like energy of *E*_l_ = 2.8 ± 0.1 eV. The observation of such dual band-gap-like behavior will be elaborated in more detail in Section 4.

Since the crystal size of CuO is very small, it is expected that the crystal lattice is significantly distorted, which can introduce a band tail, i.e., an Urbach tail,^34^ above the VB and below the CB. The width of the Urbach tail can be estimated by^35^
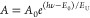
4where *A*_0_ and *E*_0_ are related to the material’s intrinsic properties, and *E*_U_ is the Urbach energy or the tail width. The red curve in Figure 1b is a semi-log plot of A versus *h*ν; from the slope of the linear fit (the blue line), *E*_U_ = 0.5 ± 0.01 eV is extracted, which is within the range of the reported values of 0.2–0.7 eV for CuO in the literature.^33^

#### 3.1.1 Transient Absorption Spectroscopy

**Figure 2:**
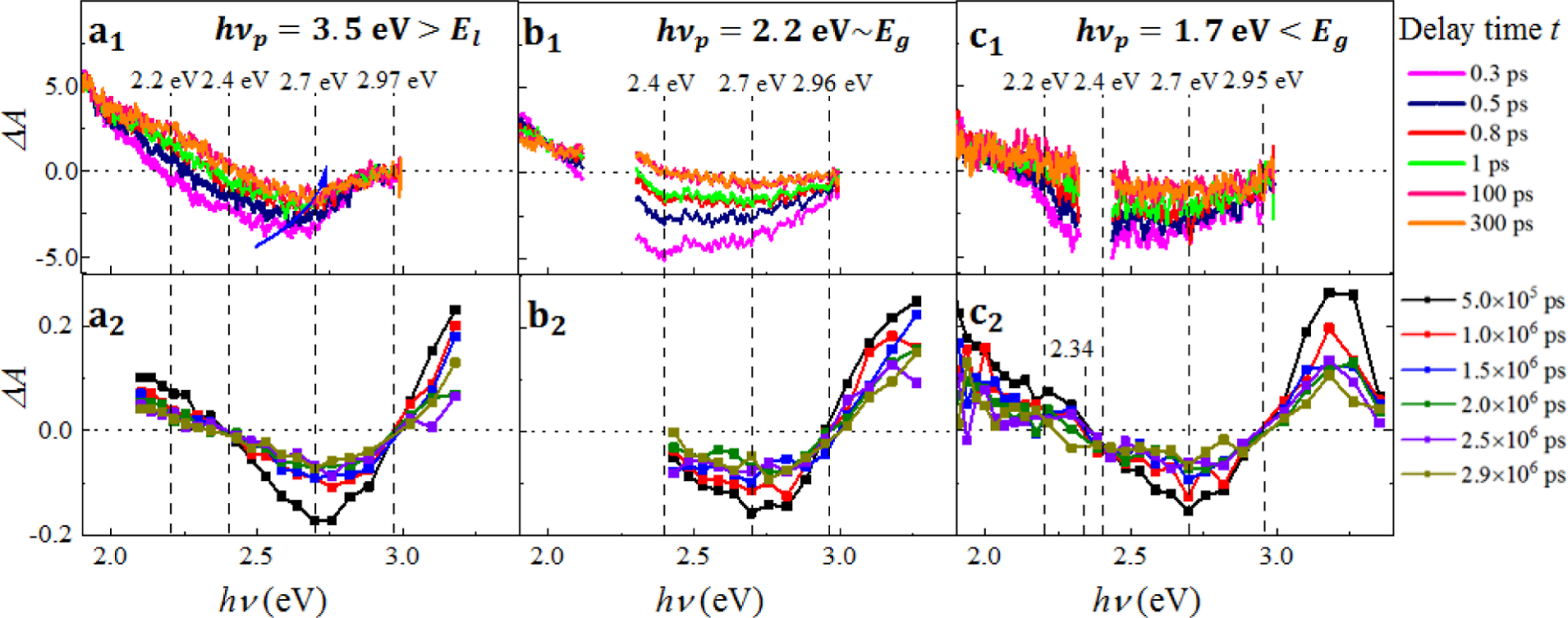
Rescaled ps- and ns-TAS spectra Δ*A*(ν,*t*)at different delay times *t* under (a) *h*ν_p_ = 3.5 eV > *E*_l_, (b) *h*ν_p_ = 2.2 eV ∼ *E*_g_, and (c) *h*ν_p_ = 1.7 eV < *E*_g_. Top row: ps-TAS spectra in the ps timescale; bottom row: ns-TAS spectra in the ns to μs timescale.

As discussed in Section 1, in order to gain a complete picture of charge dynamics, it is suggested that one needs to use different excitation energies to systematically examine the change of TAS. In the meantime, for CuO, there exists a dual band-gap-like behavior as revealed by Figure 1b. Moreover, the UV–vis spectrum in Figure S1 of the Supporting Information shows a non-zero absorption at *h*ν < *E*_g_ due to the band tail or defect absorption. Therefore, to gain a comprehensive understanding of the charge dynamics of the CuO thin film, it is necessary to investigate the TAS spectra under at least three excitation energies, *h*ν_p_ > *E*_l_, *h*ν_p_ ∼ *E*_g_, and *h*ν_p_ < *E*_g_. Both ps- and ns-TAS measurements were conducted for *h*ν_p_ = 3.5, 2.2, and 1.7 eV. To establish a quantitative comparison among TAS spectra in different timescales and *h*ν_p_, all of the obtained TAS spectra Δ*A*(ν,*t*) were rescaled by the number density of the absorbed photons, , where  is the absorption at the excitation energy *h*ν_p_ and *P* is the power per area of the excitation beam. Figure 2 shows the rescaled Δ*A*(ν,*t*) spectra as obtained by the ps-TAS (top panel) and ns-TAS (bottom panel) at different *h*ν_p_. It should be noted that the full spectra for the case of *h*ν_p_ = 2.2 eV, as shown in Figure 2b, were not accessible due to the inevitable scattering of the excitation beam.

Figure 2a_1_ shows the representative rescaled ps-TAS Δ*A*(ν,*t*) at *h*ν_p_ = 3.5 eV > *E*_l_ at different delay times *t*. Three main spectral features are observed: (1) positive Δ*A*(ν,*t*) at *h*ν < ∼2.2 eV; (2) a broad dip centered at ∼2.7 eV with an initial width of ∼0.8 eV, much broader than the other p-type semiconductors such as ZnO with a width of ∼0.2 eV;^36^ and (3) a slight blue shift of the dip position as indicated by the blue arrow in the figure. Measurements at the different locations of the sample, as shown in Figure S2, revealed that the broad dip might consist of two dips, one at 2.23 eV and the other at 2.67 eV. Regarding the time dependence of the spectra in Figure 2a_1_, as *t* increases, up to ∼1 ps, the entire spectra move up, whereas after 1 ps, they remain fairly steady. Figure 2a_2_ shows the representative ns-TAS Δ*A*(ν,*t*). The overall spectral shapes are highly comparable to those observed in the ps-TAS Δ*A*(ν,*t*), with an additional positive Δ*A*(ν,*t*) present at *h*ν > 3 eV. As *t* increases, the width and position of the broad dip remain unchanged, while both the positive and negative Δ*A* approach zero. It seems that the spectral shape of the ns-TAS Δ*A*(ν,*t*) is invariant as a function of *t*. To confirm the spectral shape changes, the time-dependent Δ*A*(ν,*t*) in Figure 2a_1_,a_2_ are normalized by the absolute value of the dip minima and are shown in Figure S3a1,a2, respectively. The shape of the normalized ns-TAS spectra is time invariant, indicating that the mechanisms for charge dynamics in the ns to μs timescale remain the same, i.e., due to the decay of charge carriers from the trap states. However, the normalized ps-TAS spectra show a strong time dependence: the positive Δ*A*(ν,*t*) at *h*ν < ∼2.2 eV becomes more pronounced at *t* ≤ 1 ps and is steady afterward. Such a time dependence of the spectral shape indicates that more than one competing mechanism dominates the charge dynamics in the ps timescale. It is noteworthy that the three samples prepared over a year have shown consistent TAS spectra (Figure S4) which confirm the reproducibility.

In the case of *h*ν_p_ = 2.2 eV ∼ *E*_g_, the ps-TAS Δ*A*(ν,*t*) in Figure 2b_1_ shows the main features observed for the previous case (Figure 2a_1_). The time dependence trends of the ps-TAS spectra are also similar to the *h*ν_p_ = 3.5 eV case (Figure 2a_1_). The spectral shape and time dependence of ns-TAS (Figure 2a_2_) shown in Figure 2b_2_ are analogous to the case of *h*ν_p_ = 3.5 eV, except for a slightly faster recovery rate in the initial few hundred ns. The normalized spectra in Figure S3b demonstrate the time invariance of the ns-TAS spectral shape and the strong time dependence of the ps-TAS spectra, consistent with the observations in the case of *h*ν_p_ = 3.5 eV.

For the *h*ν_p_ = 1.7 eV < *E*_g_ case as shown in Figure 2c, the overall spectral features and time dependence for ps-TAS and ns-TAS Δ*A*(ν,*t*) are comparable to those of *h*ν_p_ ∼ 3.5 eV and *h*ν_p_∼ 2.2 eV. Regarding the ns-TAS Δ*A*(ν,*t*), it is worth noting that since the ns-TAS spectra cover a wider range of probe energies, they reveal the existence of a peak centered at ∼3.1 eV. The normalized spectra shown in Figure S3c confirm the same observations as in both cases of *h*ν_p_ = 3.5 eV and *h*ν_p_ = 2.2 eV.

**Figure 3:**
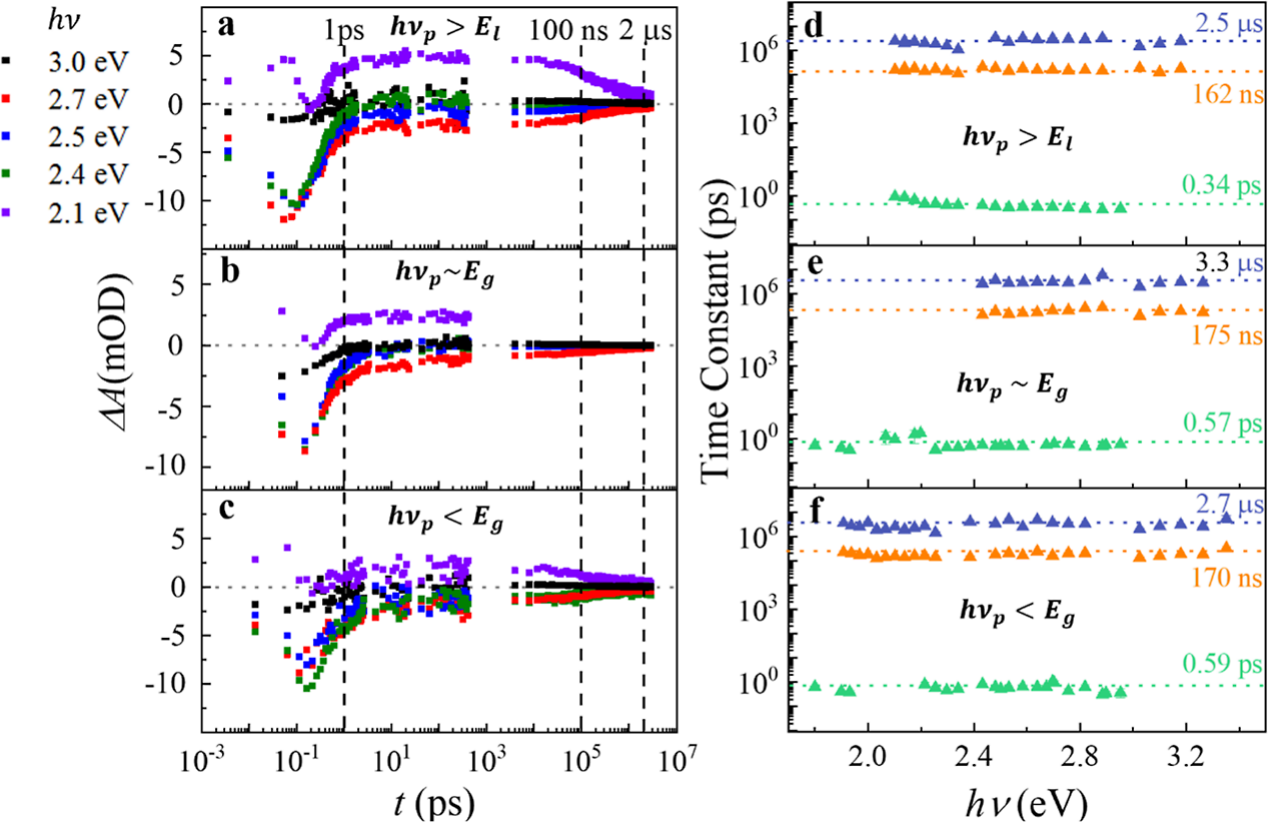
Time traces (Δ*A* versus *t* plot at a fixed *h*ν) at different *h*ν under (a) *h*ν_p_ > *E*_*l*_, (b) *h*ν_p_ ∼ *E*_g_, and (c) *h*ν_p_ < *E*_g_ and (d–f) their corresponding resolved time constants.

To better understand the time dependence of the TAS spectra in both ps and ns timescales, time traces at selected *h*ν values ranging from 2.1 to 3.0 eV and for the three different *h*ν_p_ cases from the sub-ps to μs timescale are plotted in Figure 3a–c. The *h*ν range covers two distinct spectral regions, the positive Δ*A*(ν,*t*)region at *h*ν ≤ 2.4 eV and the dip region at 2.4 eV ≤ *h*ν ≤ 3 eV. Note that since the ps and ns measurements are performed via two different setups, combining the data requires a specific strategy; herein, the ns time traces are rescaled such that the starting spectral value of the ns time trace aligns with the end spectral value of the ps time trace (at *t* ∼ 600 ps).

For *h*ν_p_ > *E*_l_ as shown in Figure 3a, beyond the ps instrument response region (*t* > 0.15 ps), the time traces at different *h*ν show a similar trend throughout the entire probed timescale: there is an initial rise at *t* ≤ 1 ps, followed by a quasi-static region in 1 ps ≤ *t* ≤ ∼100 ns, and the subsequent recovery to equilibrium at *t* > ∼100 ns. For the low probe energy region (*h*ν = 2.1) eV, Δ*A*(ν,*t*) is always positive, and the time traces initially rise, moving away from equilibrium, but plateau until a full decay in *t* ≥ 100 ns. The time trace at the low energy isosbestic point (*h*ν ∼ 2.4 eV) quickly approaches zero from a large negative value in the beginning, then fluctuates around zero. The broad dip, represented by *h*ν = 2.5 and 2.7 eV, remains negative throughout the entire timescale; the Δ*A*(ν,*t*) values increase and approach almost zero within less than 1 ps, reach a quasi-static region, and then gradually decay toward zero. The Δ*A*(ν,*t*) value at the higher energy isosbestic point (*h*ν = 3.0 eV) always fluctuates around zero. In the case of *h*ν_p_ = 2.2 eV ∼ *E*_g_, as shown in Figure 3b, the time traces are very similar to those for the *h*ν_p_ = 3.5 eV case, except that *h*ν = 3.0 eV, where Δ*A*(ν,*t*) obviously approaches zero at *t* < 1 ps. The time traces for the *h*ν_p_ = 1.7 eV < *E*_g_ case shown in Figure 3c exhibit much higher noise, but the overall time-dependent trends are very similar to those of the previous two cases.

The time traces are fitted by a linear combination of two or three exponential functions. Some representative fitting results are shown in Figures S5 and S6, respectively. It appears that the use of two exponential functions is insufficient to capture the dynamics in the ns timescale, whereas the use of three exponential functions, i.e., three time constants, results in a reasonable match with the experimental time traces. The extracted time constants τ_1_, τ_2_, and τ_3_ as a function of the probe energy *h*ν under different *h*ν_p_ are shown in Figure 3d–f. For all the three *h*ν_p_ cases, the fitted time constants are almost invariant with respect to *h*ν. For *h*ν_p_ > *E*_l_ as shown in Figure 3d, the extracted average time constants are: τ_1_ = 0.34 ± 0.13 ps, τ_2_ = 162 ± 10 ns, and τ_3_ = 2.5 ± 0.3 μs; for the *h*ν_p_ ∼ *E*_g_ case as shown in Figure 3e, τ_1_ = 0.57 ± 0.12 ps, τ_2_ = 172 ± 18 ns, and τ_3_ = 3.3 ± 0.1 μs, which are slightly higher than the corresponding values in the previous case; for *h*ν_p_ < *E*_g_ (Figure 3f), τ_1_ = 0.59 ± 0.09 ps, τ_2_ = 170 ± 19 ns, and τ_3_ = 2.7 ± 0.1 μs. Clearly, regardless of *h*ν_p_, all three time constants are quite consistent with respect to each other, which demonstrates the robustness of the results.

The fastest time constant, 0.3–0.59 ps, agrees with the reported values of 0.25–0.4 ps in the literature,^18,19,29^ which have been suggested to arise from the carrier–carrier scattering between two different energy bands. The two slow time constants around 160–175 ns and 2.5–3.3 μs are not reported in any previous literature. Nanosecond time constants are suggested to come from the recombination of charge carriers in shallow defect traps; for example, 5–12 ns was reported for TiO_2_^37^ and 3.1 ns for WSe_2_,^38^ while for the deep defect traps, 7 μs in TiO_2_^39^ and 1–10 μs in a metal halide perovskite^40^ were reported. However, another time constant of ∼2.5 ps has been suggested in the literature,^18,29^ which is not observed in the current study. This missing time constant could be rationalized through different perspectives; for instance, Born et al.^18^ used a single probe energy at 0.8 eV which is well below our probe energy range (1.9–3.0 eV) and thus does not allow for a direct comparison. Moreover, the morphology could play a role in terms of the crystallite sizes. Born et al.^18^ used CuO nanocrystals with a diameter of 50 nm and thickness of 20 nm, whereas in our study, the films are 100 nm thick with a crystallite size of 16–18 nm. Othonos et al.,^29^ on the other hand, fabricated CuO nanowires with a diameter of ≤200 nm and lengths of up to 10 μm which is much larger than those of our sample. This difference in size could induce different defect states and grain boundaries that impact the recombination time.

## 4 Energy Diagrams and Rate Equation Modeling

In order to understand the complex charge dynamics of the CuO thin film as revealed by Figures 2 and 3, a transition energy diagram with the possible transitions and corresponding rate equations are proposed based on the experimental results and previous literature reports. A simple absorption model is used to find the TAS spectra based on the solutions of the rate equations and estimated initial conditions under different *h*ν_p_. The modeled spectra capture both the spectral and time-dependent behavior of the CuO thin film from the ps to μs timescale.

### 4.1 Proposed Transition Energy Diagram and Transitions

**Figure 4:**
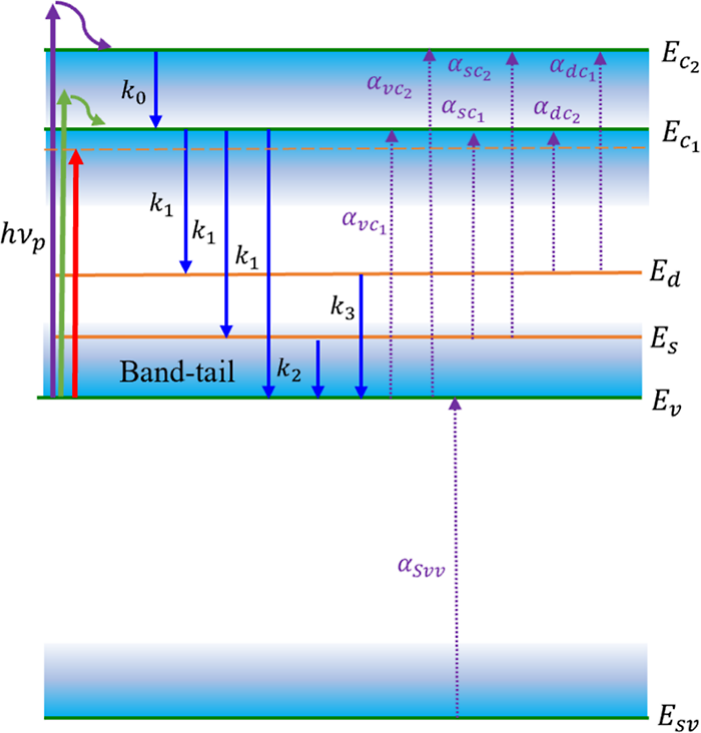
Proposed energy diagram consists of 6 energy states: , and . The blue-gradient areas show the band tails of VB and CB. The purple, green, and red arrows on the left represent three excitation cases: *h*ν_p_ > *E*_l_, *h*ν_p_ ∼ *E*_g_, and *h*ν_p_ < *E*_g_, respectively. The solid blue arrows in the middle indicate the possible transitions of electrons upon excitations and their corresponding rate constants *k*_*i*_. The purple dotted arrows represent all the possible absorption transitions, labeled with their corresponding absorption strength α_*ij*_.

The proposed energy diagram and the corresponding transitions for the three different excitation cases for the CuO thin film are shown in Figure 4. The model consists of six energy states, , and , which correspond to a SVB (sv), a VB (v), a shallow defect state (s), a deep defect state (d), the bottom of the CB (c_1_), and a higher CB energy state (c_2_), respectively. Here  and . The optical absorptions occur between the following states (dotted arrows in Figure 4): *E*_sv_ → *E*_v_, , , , , . When the electrons in the *E*_v_ state are excited to  or  states, i.e., *h*ν_p_ > *E*_l_, several decay mechanisms exist: a rapid decay from  to  with a rate of *k*_0_; the transition of electrons from  to all the lower energy states, *E*_v_, *E*_s_, and *E*_d_, all with a relatively fast rate *k*_1_; the electrons in states *E*_s_ and *E*_d_ decay back to the *E*_v_ state with rates of *k*_2_ and *k*_3_, respectively. Note that it is possible that the transitions from  to *E*_d_,  to *E*_s_, and  to *E*_v_ may occur at different times that are shorter than the resolved time constant (τ_1_ ∼ 0.34 ps), but since they are within the time resolution of the experiment, they are considered to be the same.

The CB minimum is considered to be at , which is based on the experimentally determined Tauc direct band gap of 2.2 eV (Figure 1). A higher CB state  at 2.7 eV is introduced because of the following facts: (1) in the direct band Tauc plot (Figure 1), there is another linear region which gives an intersection at 2.8 eV. This experimental result suggests a second intrinsic CB in CuO; (2) the TAS spectra always have a photobleaching dip at 2.7 eV, independent of *h*ν_p_.

Moreover, as shown in Figure S2, the broad dip consists of two different dips at 2.23 and 2.67 eV; (3) according to a cluster theory calculation on square CuO_4_ clusters within CuO as the basic units, there exists an energy state 0.5 eV higher than  due to the *b*_1g_–*e*_u_(π) transition.^41^

The two defect states, *E*_s_ and *E*_d_, are proposed based on the following reasons: (1) the existence of the two long lifetimes in the TAS analysis, τ_2_ ∼ 170 ns and τ_3_ ∼ 2.8 μs; in solid-state materials, these long lifetimes usually belong to defect state transitions.^37,42−44^ Therefore, the two long time constants most likely correspond to the kinetics involving a shallow and a deep defect state; (2) based on the TAS results, Δ*A*(ν,*t*) is positive at *h*ν < *E*_g_, which can be realized through the absorption of intragap energy states that could be defect states; (3) based on the DFT calculations, defect-induced states due to copper vacancy, *V*_cu_^–1^, and oxygen substitutional, *O*_Cu_^–1^, lie at ∼0.5 eV above the *E*_v_,^45^ which is consistent with *E*_U_; (4) previous DFT calculations have also revealed that defects like oxygen substitutional O_cu_^–2^, oxygen interstitial O_i_^–1^, and copper vacancy V_cu_^–2^ could introduce energy levels at ∼1 eV above *E*_v_.^45^ These deep defect states could correspond to *E*_d_. Therefore, in the model, we assume *E*_s_ = 0.5 eV and *E*_d_ = 1 eV relative to *E*_v_. These two defect states could originate from bulk defects such as vacancies, point defects, as well as grain and grain boundaries. It is worth noting that the difference in grain boundary characteristics can lead to a variation in defects properties. For instance, it has been shown that an increase in grain boundary area is correlated with longer recombination lifetimes in Cu_2_S thin films.^46^ For NiO^47^ and perovskite solar cells,^48^ the grain boundaries have a detrimental impact on the charge separation by mediating the electron and hole recombination. The grain boundaries can also induce surface states in the band gap of Si/SiO_*x*_, resulting in a red shift in the ground state bleaching as crystalline domain sizes decrease.^49^

The SVB, *E*_sv_, is introduced based on the following evidence: (1) the ns-TAS spectra reveal the appearance of a positive Δ*A*(ν,*t*) at *h*ν > 3 eV (see Figure 2). Such an energetic peak should correspond to a transition between two intrinsic states which has also been attributed to the transition from the SVB to the VB in the literature;^18,29^ (2) the steady-state optical absorption (Figure S1) increases monotonically with *h*ν, indicating strong high energy transitions above 3 eV; (3) a photoelectrochemical study shows that the VB of CuO consists of two separated VBs, a top VB made from 3d orbitals of Cu^+2^ and an SVB due to an oxygen-2p-type band located at ∼2 eV below *E*_v_.^50^ In our ns-TAS measurements, this positive peak appears at 3.2 eV, which could belong to the SVB to VB absorption; thus, in the model, the SVB is set to *E*_sv_ = −3.2 eV.

For the non-equilibrium transition of electrons, it is assumed that *k*_0_ > *k*_1_ ≫ *k*_2_ > *k*_3_, where *k*_*j*_ (*j* = 0, 1, 2, 3) is the rate constant of the corresponding transition, which is defined as  (where *j* = 0, 1, 2, 3) (in Section 4.3 we will show that these rate constants correspond to the experimentally obtained time constants). In the case of photoexcitation to , in relaxation to , electrons undergo coherent processes, such as momentum scattering, carrier–carrier scattering, intravalley scattering, and hole-optical phonon scattering, all of which occur in ≤∼200 fs;^51−53^ hence, a single ultrafast rate constant *k*_0_ is defined to account for these effects. Note that the timescale of these processes lies within the instrument response time of the measurements; thus, *k*_0_ is not resolvable in this study. In the literature, the trapping timescale of metal oxides, such as BiVO4,^54^ SnO_2_,^43^ and TiO_2_,^44^ is reported to be few ps when electrons fall from the CB minimum to the defect/trap states, . Thus, the second fastest rate constant is associated with the decay of  to the low-lying energy states and is denoted as *k*_1_ in the model. Moreover, the timescale of the recombination of the charge carrier in shallow traps is reported to be 5–12 ns for TiO_2_^37^ and 3.1 ns for WSe_2_,^38^ while the deep traps have a lifetime of 7 μs for TiO_2_^39^ and 1–10 μs in a metal halide perovskite;^40^ moreover, in CuO, a time constant of >50 ps is associated with the recombination of electrons and holes.^18,19^ Thus, in the current study, the experimentally observed τ_2_and τ_3_ are assumed to originate from the de-trapping of electrons from the shallow and deep trap states, and the decay rates are denoted by *k*_2_ and *k*_3_, respectively. It is worth noting that there is no observable transition between the defect states because of their low density and spatially separated locations in thin films.

### 4.2 Rate Equations and Solutions

Due to different *h*ν_p_, three sets of rate equations can be established based on the proposed transition energy diagram and are summarized in Table 1 with their corresponding initial conditions. Here, Δ*n*_i_(*t*) (i = v,s,d,c_1_,c_2_) is the nonequilibrium electron population of the energy level i at time *t* after excitation, *k*_*j*_(*j* = 0, 1, 2, 3) is the rate constant of the corresponding transition, and Δ*n* denotes the total number density of the photoexcited electrons by the excitation beam. For the experimental spectra as shown in Figure 2, since the spectra were normalized according to the absorbed photon flux at *h*ν_p_ (assuming an effective quantum efficiency of 100%, i.e.*,* each absorbed photon generates an electron–hole pair), the same Δ*n* is used for all the three *h*ν_p_ cases. For the case of *h*ν_p_ ∼ *E*_g_ and *h*ν_p_ < *E*_g_, both the rate equations and the initial conditions are exactly the same since for *h*ν_p_ < *E*_g_, we assume that electrons from the VB tails are excited to , and the VB and VB tail are treated as a single state. Such an assumption is based on the multiple experimental evidence of measured IPCE (incident photon conversion efficiency) spectra for CuO, where the incident photon with an energy lower than the band gap can still introduce a photocurrent.^55,56^ However, for *h*ν_p_ > *E*_l_, both the initial conditions and the rate equations are different from the other two cases since an additional energy state, , is involved in the charge dynamics.

**Table 1:** Rate Equations and Initial Conditions for the 3 Excitation Cases

	*h*ν_p_ > *E_l_*	*h*ν_p_ ∼ *E*g, *h*ν_p_ < *E*g
rate equations	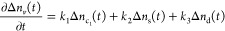	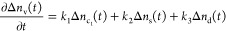
	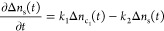	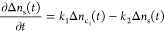
	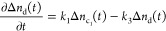	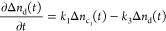
	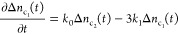	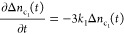
	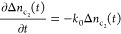	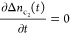
initial conditions	Δ*n*_v_(*t* = 0) = −Δ*n*	Δ*n*_v_(*t* = 0) = −Δ*n*
	Δ*n*_s_(*t* = 0) = 0	Δ*n*_s_(*t* = 0) = 0
	Δ*n*_d_(*t* = 0) = 0	Δ*n*_d_(*t* = 0) = 0
	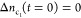	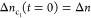
	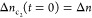	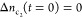

The complete solutions of the rate equations are provided in Table S1 of the Supporting Information. As expected, the solutions for *h*ν_p_ ∼ *E*_g_ and *h*ν_p_ < *E*_g_ cases are identical, while the solutions for the *h*ν_p_ > *E*_l_ case are significantly different. For *h*ν_p_ > *E*_l_,  is dominated by *k*_0_, and all other solutions for  contain an exponential decay function of , while for the other two cases, . Since *k*_0_ > *k*_1_ ≫ *k*_2_ > *k*_3_, at a sufficiently long time *t*,  and thus, it is interesting to notice that the solutions eventually become the same for both the *h*ν_p_ > *E*_l_ case and the *h*ν_p_ ∼ *E*_g_ or *h*ν_p_ < *E*_g_ case; i.e., at a sufficiently long initial delay time, the solutions of all the cases are exactly the same.

**Figure 5:**
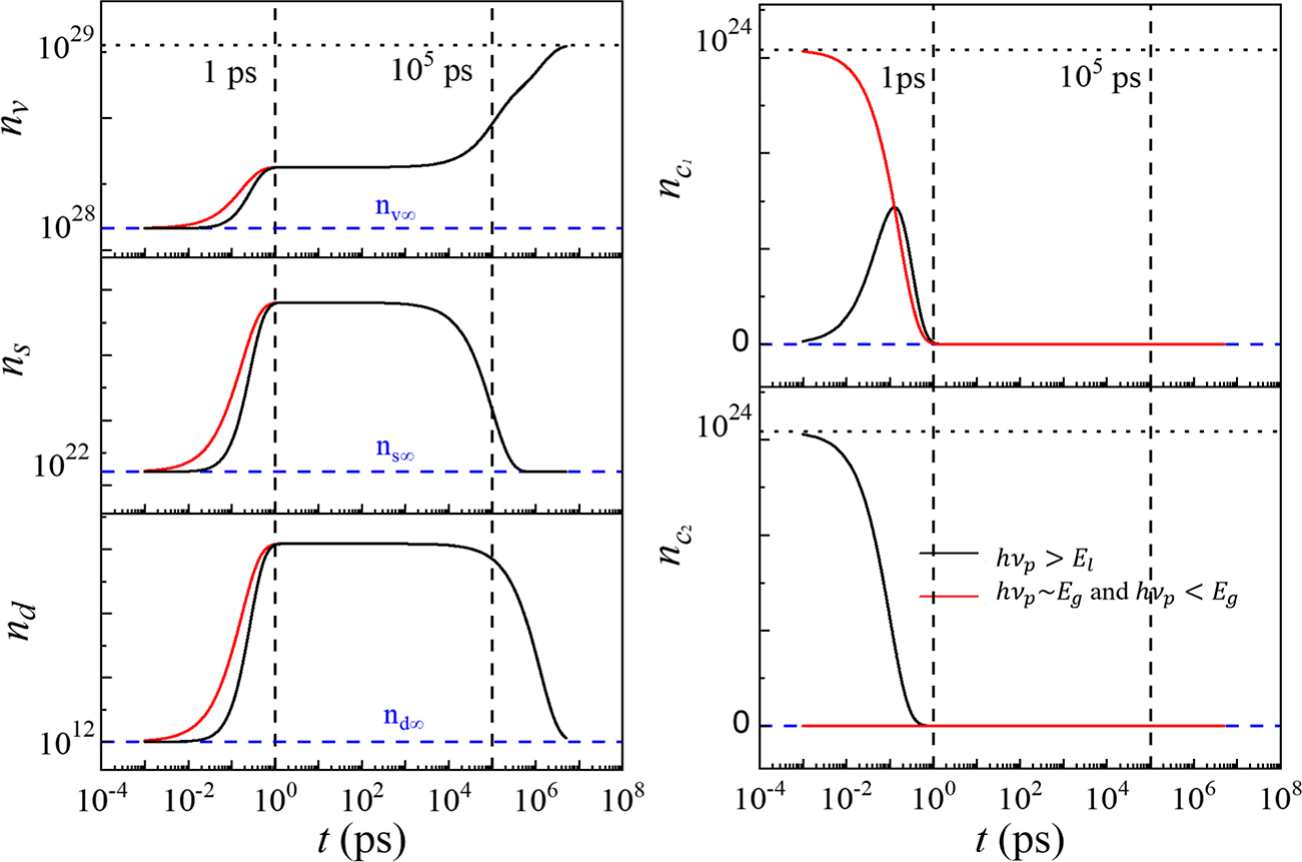
Total electron population at different energy states under 3 excitations. Black: *h*ν_p_ > *E*_l_, red: *h*ν_p_ ∼ *E*_g_ and *h*ν_p_ < *E*_g_. The equilibrium populations, *n*_v∞_, *n*_s∞_, and *n*_d∞_, are denoted by the horizontal blue dashed lines. The initial population of the CBs are assumed to be zero in the model.

Based on the lattice properties of the CuO crystal, the equilibrium population parameters are estimated in Section S6 of the Supporting Information. The total electron population, *n*_i_ = Δ*n*_i_ + *n*_i∞_ (where i = v,s,d,c_1_,c_2_, and *n*_i∞_ is the equilibrium population), for different energy states can be obtained and are plotted in Figure 5, where the equilibrium populations, *n*_v∞_,*n*_s∞_, and *n*_d∞_, are denoted by the horizontal blue dashed lines. Note that  in the calculation.

The populations from the solution of the rate equations are plotted in Figure 5. The time constants τ_1_ = 0.5 ps, τ_2_ = 170 ns, and τ_3_ = 2.8 μs are taken as the average of the fitted values in the 3 excitation cases from Figure 3d–f and τ_0_ is set to 0.1 ps. Figure 5 reveals two common features regardless of the *h*ν_p_: (1) the entire timescale can be divided into 3 stages: stage#1 ultra-fast stage (*t* < 1 ps), where the populations of the electrons in all the energy states change rapidly; stage #2 quasi-static stage (1 ps < *t* < 100 ns), where all the populations remain almost constant; stage #3 recovery stage (*t* > 100 ns), where the populations approach the equilibrium values. These stages are consistent with the three stages observed experimentally (Figure 3). (2) for *t* < 1 ps, *n*_v_, *n*_s_, and *n*_d_ increase rapidly to a quasi-equilibrium value, whereas  and  decrease to 0. These common behaviors reflect the intrinsic charge dynamics governed by the three time constants; upon excitations, all the electron populations are driven away from equilibrium, and the dynamics of the electrons are governed by the smallest time constant at small *t*. However, since there is no transition in the ps to ns timescale, *n*_v_, *n*_s_, and *n*_d_ would remain unchanged until a later time; when defect states release the trapped electrons to the VB, *n*_s_, *n*_d_, and *n*_d_ start to acquire their equilibrium values. There are two significant differences between the case of *h*ν_p_ > *E*_l_ and the cases of *h*ν_p_ ∼ *E*_g_, and *h*ν_p_ < *E*_g_: (1) for *t* < 1 ps, the rise of *n*_v_, *n*_s_, and *n*_d_ is faster for the cases of *h*ν_p_ ∼ *E*_g_ and *h*ν_p_ < *E*_g_ compared to that of *h*ν_p_ > *E*_l_; (2) the behaviors of  and  are very different.  shows a peak for the *h*ν_p_ > *E*_l_ case since in the first few hundred femtoseconds, the hot electrons in  relax to , causing a spike in , and a sharp decrease in . The observed difference between the *h*ν_p_ > *E*_l_ excitation case and those of *h*ν_p_ ∼ *E*_g_ and *h*ν_p_ < *E*_g_ is because there is no relaxation from  to  in the latter two cases, and hence, the decay of the electrons from  to *E*_v_, *E*_s_, and *E*_d_ occurs instantaneously after the photoexcitation; so, compared to the case of *h*ν_p_ > *E*_l_, *n*_v_, *n*_s_, and *n*_d_ undergo a steeper rise in the beginning. The populations for the longer timescale are identical in all three cases since the transitions in the ns to μs timescale are identical.

### 4.3 Modeling of Transient Absorption Δ*A*(ν,*t*)

Based on the solutions of the rate equations, the optical absorption *A*_i*j*_(*t*) from the *i*th state to the *j*th state with a resonance frequency  (*E*_*j*_ and *E*_i_ are the energy of the *j*th and *i*th energy levels, respectively) can be written as
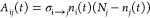
5where  is the absorption cross section; *n*_i_(*t*) and *n*_*j*_(*t*) are the population of the initial and final states at time *t*, respectively; and *N*_*j*_ is the total number of available states in *E*_*j*_. Ideally, the absorption spectrum can be written as
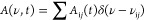
6where the summation runs over all the possible allowed absorption transitions and  is the δ-function, resembling the discretized spectral lines. However, due to carrier scattering, thermal fluctuations, and other spectral line broadening mechanisms, the actual spectral line shape is not a δ-function but usually can be represented by a Lorentzian shape or other spectral shapes. Here, we use the Lorentzian spectral shape *S*_i*j*_(ν)
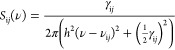
7where γ_i*j*_ is the oscillation damping factor which determines the absorption peak widths, varying for different transitions. Then, according to eq 1, the TAS spectrum is determined. Based on Figure 4, there are seven possible absorptions that can contribute to the final transient absorption spectra, and the overall Δ*A*(ν,*t*) can be written theoretically as
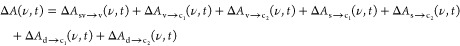
8

Note that there is no absorption between the shallow and deep defect states as they are spatially separated, and a direct transition is less probable. Considering eqs 1 and 5–8, Δ*A*(ν,*t*) is given by
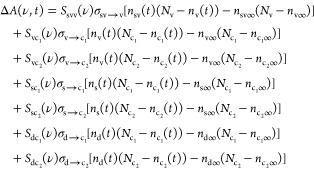
9

Equation 9 includes all of the absorption transitions shown in Figure 4. However, due to the finite spectral width, each resonant absorption could only significantly contribute to the absorption in a wavelength range near its resonant frequency, i.e., ν_i*j*_ – 2γ_i*j*_ ≤ *h*ν ≤ ν_i*j*_ + 2γ_i*j*_, which means at a particular *h*ν, not all the 7 absorption transitions in eq 9 need to be considered. As described in the next section, tihe γ_i*j*_ values are smaller than 0.5 eV, so only a limited number of resonant absorption peaks near *h*ν need to be considered. For instance, Section S7 of the Supporting Information shows the explicit expressions for Δ*A*(ν,*t*) at *h*ν = 2.2 eV (eq S4) and *h*ν = 2.7 eV (eq S6) in the *h*ν_p_ ∼ *E*_g_ excitation case; only 4 or 2 resonant transitions are considered. By further considering *k*_1_ ≫ *k*_2_ > *k*_3_, the final expression of Δ*A*(ν,*t*) only depends on the three time-dependent exponential functions, , , and , respectively. The results show that independent of the spectral region, Δ*A*(ν,*t*) can be expressed as the sum of 3 time-dependent exponential functions with time constants which are directly related to the experimentally resolved time constants. Thus, *k*_*j*_ = 1/τ_*j*_ is validated.

Clearly, based on the solutions of the rate equations, once both the values of  and γ_i*j*_ for each absorption transitions are determined, one should be able to model Δ*A*(ν,*t*). In order to obtain these two sets of parameters, a spectral analysis strategy based on the experimental ps-TAS Δ*A*(ν,*t*) for the *h*ν_p_ = 3.5 eV > *E*_l_ case is employed, and the details are explained in Section S8 of the Supporting Information. The results are presented in Figures S5 and S6, and the corresponding video for the spectral fittings is provided as Movie M1. The plots of the fitted energy levels, *E*_i_, and damping constants, γ_*ij*_, versus decay time *t* (Figure S7a,b) show that these two sets of parameters are invariant with respect to *t*. Further analysis of the absorption cross sections, , based on the solutions of the rate equations (Table S1) shows that all , corresponding to the different absorption transitions in Figure 4, converge to constant values at *t* ≥ 1 ps, and those values are used in the spectral modeling. All the parameters used to model the Δ*A*(ν,*t*) for different *h*ν_p_ cases and from ps to μs timescales based on eq 9, and the solutions of the rate equations (Table S1), are listed in Table S2.

**Figure 6:**
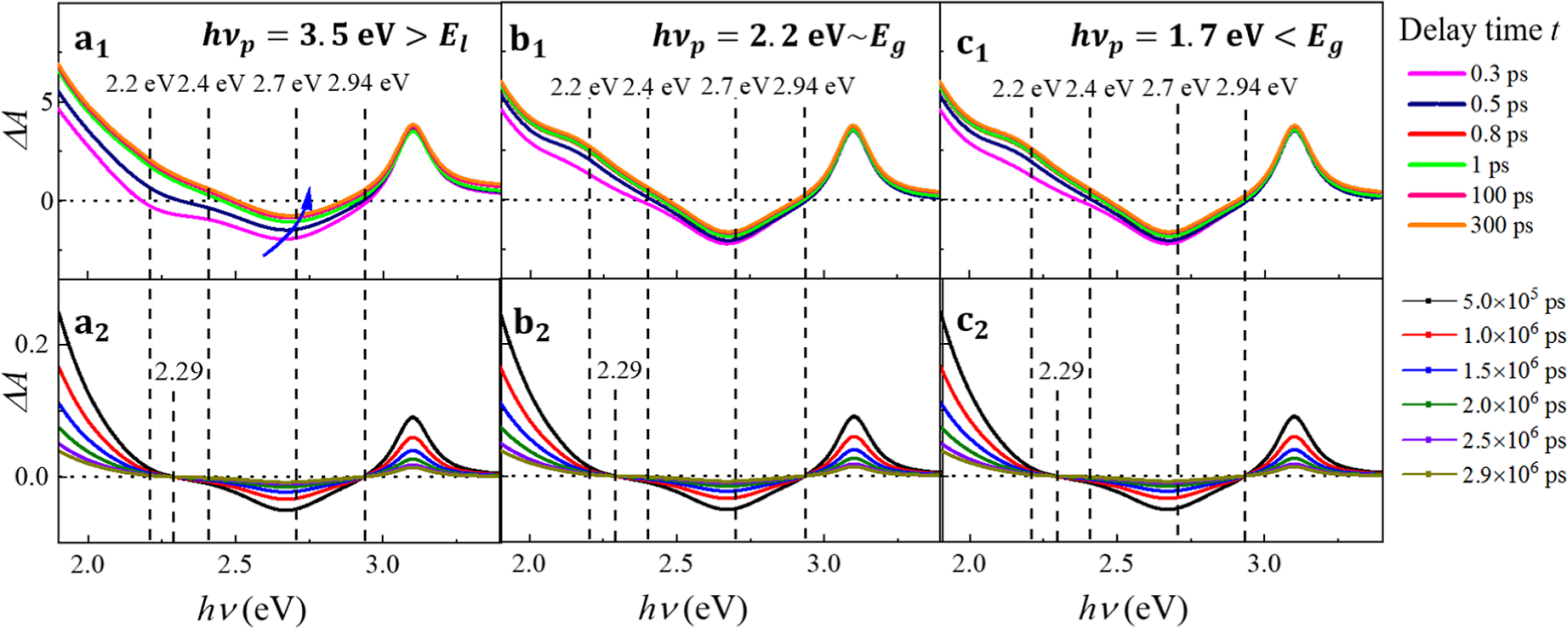
Modeled ps- and ns-TAS spectra Δ*A*(ν,*t*)at different delay times *t* under (a) *h*ν_p_ = 3.5 eV > *E*_l_, (b) *h*ν_p_ = 2.2 eV ∼ *E*_g_, and (c) *h*ν_p_ = 1.7 eV < *E*_g_. Top row: ps-TAS spectra; bottom row: ns-TAS spectra.

Figure 6 shows the modeled TAS spectra Δ*A*(ν,*t*) under the 3 excitation cases based on eq 9, the solution of the rate equations, and parameters in Tables S1 and S2. For all three cases, the Δ*n* values are assumed to be the same, 2.5 × 10^24^ m^–3^. In the case of *h*ν_p_ > *E*_l_, as shown in Figure 6a_1_, similar to the experimental results (Figure 2a_1_), four main spectral features are observed in ps-TAS: (1) positive Δ*A*(ν,*t*) at *h*ν < ∼2.2–2.4 eV; (2) positive Δ*A* at *h*ν > ∼ 2.9 eV and a peak at 3.1 eV; (3) a broad dip at ∼2.67 eV with an initial width of ∼0.8 eV; and (4) a slight blue shift of the dip position as indicated by the blue arrow in the figure. As *t* increases up to 0.5 ps, the entire spectra move up, then remain fairly steady, consistent with the experimental Δ*A*(ν,*t*). For the ns-TAS spectra as shown in Figure 6a_2_, three features are observed: (1) positive Δ*A*(ν,*t*) at *h*ν > 3 eV and a peak at 3.1 eV; (2) positive Δ*A*(ν,*t*) at *h*ν < 2.3 eV; and (3) a broad dip at 2.3 eV < *h*ν <3 eV, which is centered at ∼2.7 eV. Other experimental features are also captured, including the time invariance of the dip width and position, and all the Δ*A*(ν,*t*) values approach zero with time. The normalized Δ*A*(ν,*t*) spectra are plotted in Figure S9a, similar to the normalized experimental spectra shown in Figure S3a; the time-dependent trends in spectral shape are the same: for ps-TAS spectra, the spectral shape changes significantly, especially in the *h*ν < 2.0–2.4 eV region, and all the normalized positive Δ*A*(ν,*t*) values increase with *t*; for ns-TAS, the normalized spectra are invariant with time.

The ps-TAS Δ*A*(ν,*t*) for the *h*ν_p_ ∼ *E*_g_ case, as shown in Figure 6b_1_, has quite similar features to the experimental results (Figure 2b_1_), with positive Δ*A*(ν,*t*) values at *h*ν < ∼2.0–2.4 eV, and a broad negative dip centered at ∼2.67 eV. The only difference is the distinguished peak at 3.1 eV. The time dependence of ns-TAS spectra is similar to those shown in Figures 2b_2_ and 6a_2_. The normalized ps-TAS and ns-TAS spectra shown in Figure S9b are also consistent with those observed experimentally (Figure S3b) and for the *h*ν_p_ > *E*_l_ case. For the *h*ν_p_ < *E*_g_ case as shown in Figure 6c_1_,c_2_, since the solutions for the rate equations are exactly the same for the *h*ν_p_ ∼ *E*_g_ case, the time-dependent TAS spectral behaviors are identical to those shown in Figure 6b_1_,b_2_.

**Figure 7:**
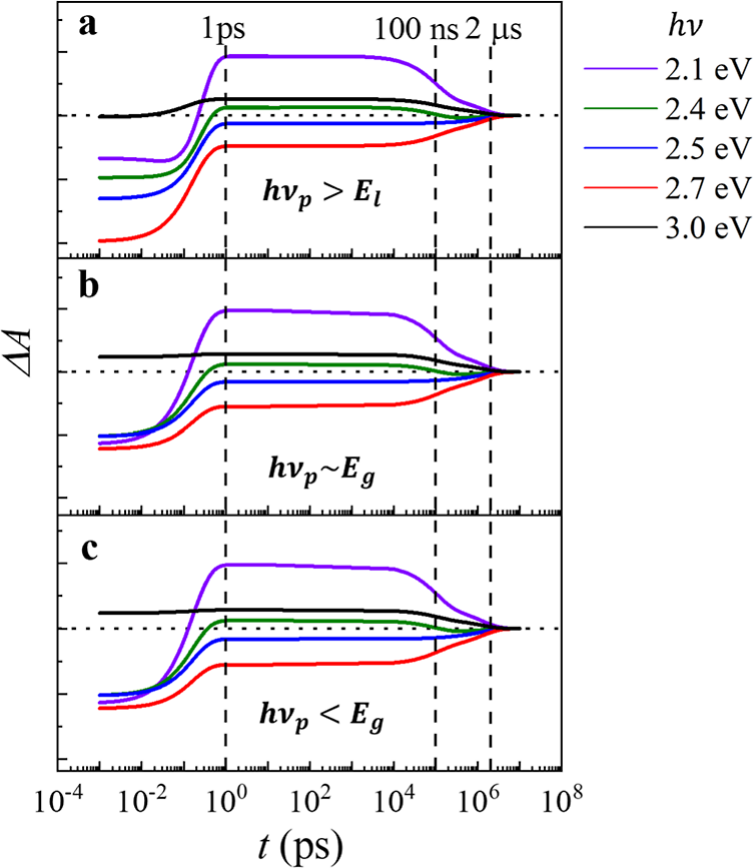
Time traces of the model spectra at different probe energies (*h*ν) under (a) *h*ν_p_ > *E*_l_, (b) *h*ν_p_ ∼ *E*_g_, and (c) *h*ν_p_ < *E*_g_. The dashed lines indicate the approximate time to divide the whole timescale into three stages.

The time traces of the modeled spectra for different excitation cases at selected *h*ν values ranging from 2.1 to 3.0 eV are plotted in Figure 7. For all *h*ν_p_ cases, the trends of different time traces are similar at different *h*ν: there is an initial rise in Δ*A*(ν,*t*) within the first 1 ps, then the time traces plateau within 1 ps ≤ *t* ≤ ∼10 ns, and finally Δ*A*(ν,*t*) approaches zero at *t* ≥ ∼10 ns. Similar to the experimental time traces, at the low energy region, *h*ν < 2.4 eV, which is represented by *h*ν = 2.1 eV, Δ*A*(ν,*t*) undergoes a rise and moves away from equilibrium but returns back to zero in the μs timescale. The time trace near the low energy isosbestic point, *h*ν ∼ 2.4 eV, follows the same trend. Δ*A*(ν,*t*) values in the broad dip region, represented by *h*ν = 2.5 and 2.7 eV, remain negative throughout the entire time range, as expected, and approach zero (equilibrium) in the μs timescale. In the high energy region, as represented by *h*ν = 3.0 eV, Δ*A*(ν,*t*) slightly increases at *t* ≤ 1 ps for the *h*ν_p_ > *E*_l_ case, plateaus, and recovers to zero in the μs timescale. For the *h*ν_p_ ∼ *E*_g_ and *h*ν_p_ < *E*_g_ cases, the Δ*A*(ν,*t*) value at *h*ν = 3.0 eV remains almost a constant for *t* ≤ 10 ns and then approaches zero at *t* > 100 ns. The reason for this difference is that in the case of *h*ν_p_ > *E*_l_, the electrons are initially excited to  and decay to  within 0.1 ps; thus, the transitions to the low-lying energy levels are delayed compared to the other two cases.

The modeled spectra Δ*A*(ν,*t*) not only successfully capture the main features of the experimental TAS spectra but can also be directly compared to the experimental spectra after rescaling by Δ*n*. Movies M2, M3, and M4 show the direct comparison of the modeled spectra (blue curves) and the experimental spectra (black curves), from the sub-ps to μs timescale for three *h*ν_p_ cases. All the contributions due to absorptions from different intrinsic transitions marked in Figure 4 are shown as dashed red curves for ps-TAS. Regardless of the *h*ν_p_, two general trends are observed: when *t* < 1 ps, there are relative large discrepancies between the modeled spectra and the experimental spectra, especially in *h*ν < 2.4 eV; when *t* > 1 ps, the modeled spectra and the experimental spectra match very well. These results show that our model (Figure 4) and the solution of the rate equations are reasonable to explain both the spectral shape and the time dependence of the experimental TAS for relatively long time delays (*t* > 1 ps). But there may be some other mechanisms that are missing in the short time period. Physically at *t* < 1 ps, many electrons from VB are excited to CB, and there is a significant amount of photo-generated free electrons in the material. Thus, one possible contribution for the discrepancies observed in Movies M2, M3, and M4 is the free-electron absorption (FEA), which can be described by^57^
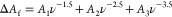
10where Δ*A*_f_ is the TAS due to the FEA and *A*_*i*_ (*i* = 1, 2, 3) are the related constants. The first power-law term in eq 10 is due to the electron scattering by acoustic phonons, the second power-law term comes from electron scattering by optical phonons, and the last term is due to the scattering by ionized impurities.^57^ The effect of the FEA in the ps-TAS spectra can be estimated by fitting the difference between the modeled spectra and corresponding experimental spectra at *h*ν < 2.4 eV for *t* ≤ 1 ps by eq 10, and the results are presented in Movies M5, M6, and M7. Based on the fits, the *A*_*i*_ coefficients in eq 10 are estimated as, *A*_1_ = *A*_2_ = 0, regardless of the excitation energy, and *A*_3_ = 7.6 × 10^–10^, 3.4 × 10^–10^, and 0.7 × 10^–10^ for *h*ν_p_ = 3.5, 2.2, and 1.7 eV, respectively. The estimated Δ*A*_f_ is then added back to the corresponding modeled spectra at *t* < 1 ps. Movies M8, M9, and M10 show the comparison of the FEA-corrected spectra and experimental TAS spectra. Clearly, the agreement between the modeled spectra and experimental spectra is improved.

## 5 Conclusions

In this work, we have presented a thorough understanding of the charge dynamics of a CuO thin film by reproducing the experimental TAS Δ*A*(ν,*t*) with a model based on rate equations which successfully capture the main observed experimental features. This study implies that, in order to gain a complete insight into the charge dynamics, (1) TAS measurements should be performed in a broad timescale and excitation energy range, and (2) the TAS analysis should be accompanied by appropriate models, i.e., rate equations based on reasonable assumptions.

The TAS experiments were done in a broad timescale, from ps to μs, and under three different excitation energies: above, near, and below the band gap. Regardless of the excitation energy, the main spectral features remained consistent with time. Δ*A*(ν,*t*) was positive at low and high energies, while there was a very broad negative dip, centered at ∼2.7 eV which was proven to consist of two overlapping dips at 2.2 and 2.7 eV by performing the TAS measurements at a different spot of the sample and a careful analysis of the UV–vis spectrum. To have a closer look at the spectral changes with time, the ps- and ns-TAS spectra were normalized by the value of the absolute minimum. The results showed that in the normalized ps-TAS Δ*A*(ν,*t*), the spectra slightly changed with time at low energies, while the normalized ns-TAS Δ*A*(ν,*t*) was time independent, indicating that the mechanism of the charge dynamics remains the same in the ns to μs timescale. To gain a better understanding of the charge dynamics, the time traces were analyzed at different probe energies. Independent of the excitation energy, three regions were identified: (1) ultra-fast region (*t* < 1 ps); (2) quasi-static region (1 ≤ *t* ≤ 100 ns); and (3) recovery region (*t* < 100 ns), where in the ultra-fast region, time traces quickly increased, then plateaued in the quasi-static region, and approached zero (equilibrium) in the recovery region. Regardless of the excitations, three time constants, τ_1_ ∼ 0.34–0.59 ps, τ_2_ ∼ 162–175 ns, and τ_3_ ∼ 2.5–3.3 μs, were resolved, indicating the dominating charge dynamics in very different timescales.

Based on the TAS Δ*A*(ν,*t*), UV–vis, and previous studies, a compelling transition energy diagram was proposed which consists of a higher energy CB, ; shallow and deep defect states, *E*_s_ and *E*_d_, respectively; and an SVB state. The higher CB, , was proposed based on the observation of a double dip in the ps-TAS Δ*A*(ν,*t*) as well as the analysis of the Tauc plot. The defect states were introduced because of the existence of grain boundaries in the crystal that break the periodicity of the lattice and induce defect states. The existence of such defect states has also been supported in previous DFT studies. The SVB was considered due to the observation of an energetic peak in the ps- and ns-TAS Δ*A*(ν,*t*) as well as the energetic high intensity absorption in the UV–vis spectra. A decay mechanism was proposed such that upon excitation by the ultra-fast pulse above , the electrons decay very quickly (*t* < 100 fs) to  and then decay to the defect states and the VB within 0.5 ps. The de-trapping from the shallow and deep defect states to the VB was considered to occur in ∼170 ns and 2.8 μs, respectively, which were found from the averages of the long time constants in the 3 different excitation energies.

The experimental TAS Δ*A*(ν,*t*) were modeled by solving the rate equations based on the proposed energy diagram and the corresponding transitions, and a simple expression for the Δ*A*(ν,*t*) was defined by assuming a Lorentzian function for the spectral shape of the transitions. The parameters of the model were found by fitting the spectra of the *h*ν_p_ = 3.5 eV excitation case to the derived Δ*A*(ν,*t*). The modeled time-dependent spectra were explicitly compared to the experimental TAS Δ*A*(ν,*t*) as shown by the videos which indicated a very good agreement at *t* > 1 ps. As a plausible mechanism to explain the inconsistencies at *t* < 1 ps, free-electron absorption was investigated and applied to the model which improved the agreement and thus can be considered a contributing mechanism in the very short timescale.

The proposed approach in this study, i.e., using a broad timescale and different excitation energies in the TAS measurements, and the complementary model based on the rate equations successfully elucidated the charge dynamics of the CuO thin film. However, one should note that the model is only valid within the introduced assumptions. For instance, the band tails are considered as single energy states instead of continuous bands, and all the decay mechanisms, i.e., recombination, are assumed to be of the first order. Considering these assumptions, the model can be generalized to other materials given that it is necessary to have a priori knowledge about the optical properties, i.e., band gap, of the system as well as the intra-gap energy states, i.e., defect states.

A future study could be thickness-dependent measurements to evaluate the effect of film thickness on the dynamics of photo-induced charge dynamics. This is because, in addition to electron–hole recombination, carrier diffusion within the film is also affected by the film’s thickness. Moreover, the variation of pump light intensity across the film’s depth can create a depth-dependent concentration gradient of photo-induced electrons, which would be more prominent in thicker films.

## Supporting Information Available

Experimental spectral fittings of ps-TAS spectra for the 3.5 eV case to extract the simulation parameters (MP4)
Comparison of the simulated TAS spectra versus the experimental spectra in the ps to μs timescale for the *h*ν_p_ = 3.5 eV case (MP4)
Comparison of the simulated TAS spectra versus the experimental spectra in the ps to μs timescale for the *h*ν_p_ = 2.2 eV case (MP4)
Comparison of the simulated TAS spectra versus the experimental spectra in the ps to μs timescale for the *h*ν_p_ = 1.7 eV case. (MP4)
Free electron absorption analysis for the *h*ν_p_ = 3.5 eV case (MP4)
Free electron absorption analysis for the *h*ν_p_ = 2.2 eV case (MP4)
Free electron absorption analysis for the *h*ν_p_ = 1.7 eV case (MP4)
Comparison of the FEA-corrected simulated TAS spectra versus the experimental spectra in the ps to μs timescale for the *h*ν_p_ = 3.5 eV case (MP4)
Comparison of the FEA-corrected simulated TAS spectra versus the experimental spectra in the ps to μs timescale for the *h*ν_p_ = 2.2 eV case (MP4)
Comparison of the FEA-corrected simulated TAS spectra versus the experimental spectra in the ps to μs timescale for the *h*ν_p_ = 1.7 eV case (MP4)
Steady-state absorption spectrum, double dip revealed in ps-TAS measurements, ps-TAS spectra at high energy, normalized TAS spectra, examples of time trace analysis, solution of rate equations, estimation of the initial electron populations, expression of Δ*A*(ν,*t*), estimation of σ_i→*j*_ and γ_i*j*_, normalized model spectra, and description of the videos (PDF)
