# Supplementary material for: Charge Dynamics of a CuO Thin Film on Picosecond to Microsecond Timescales Revealed by Transient Absorption Spectroscopy
Source: ACS Appl Mater Interfaces. 2023 Mar 30;15(14):18414–26. doi: 10.1021/acsami.2c22595 (PMC10103062; doi:10.1021/acsami.2c22595)
Supplement: Supplementary file 11 — am2c22595_si_011.pdf [file am2c22595_si_011.pdf]

Supporting Information

The Charge Dynamics of a CuO Thin Film on Pico-second to Micro-second Timescales Revealed by Transient Absorption Spectroscopy

Mona Asadinamin,[1] Aleksandar Živkovic[2], Susanne Ullrich,[*1] Henning Meyer,[*1] and Yiping Zhao[*1]

[1] *Department of Physics and Astronomy, University of Georgia, Athens, Georgia 30602, USA*
[2] *Department of Earth Sciences, Utrecht University, Princetonlaan 8a, 3548CB Utrecht, The Netherlands*

Corresponding Authors

Susanne Ullrich- Department of Physics and Astronomy, University of Georgia, Athens, Georgia 30605, United States; Email: ullrich@uga.edu

Henning Meyer- Department of Physics and Astronomy, University of Georgia, Athens, Georgia 30605, United States; Email: hmeyer@uga.edu

Yiping Zhao- Department of Physics and Astronomy, University of Georgia, Athens, Georgia 30605, United States; Email: zhaoy@uga.edu

S1. Steady-state absorption spectrum

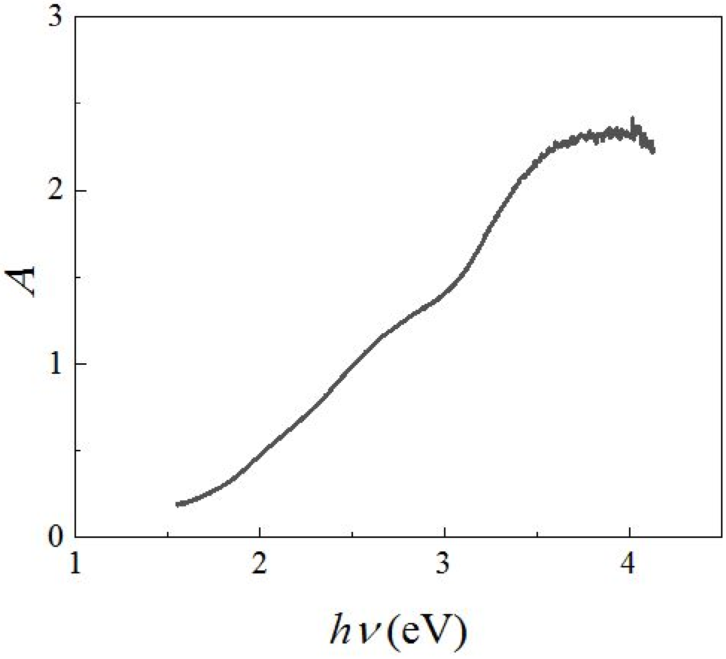

Fig. S1 A typical UV-Vis absorption spectrum of the CuO thin film.

S2. Double dip revealed in ps-TAS measurements

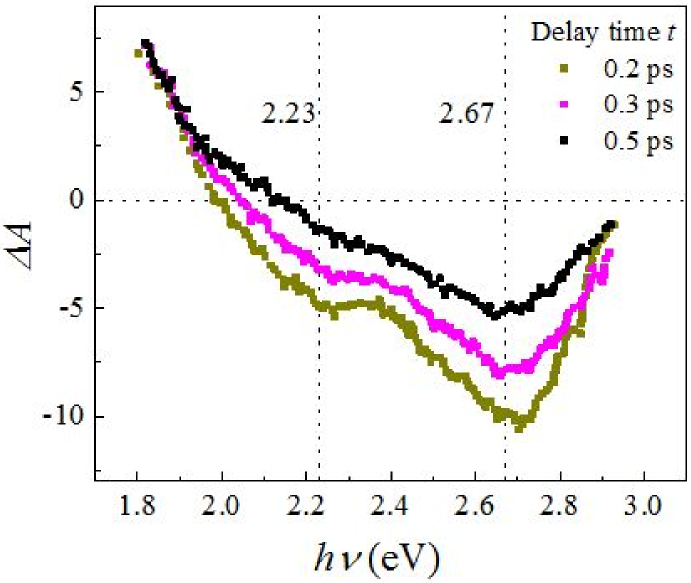

Fig. S2 The ps-TAS spectra at a different location of the CuO thin film sample. The spectra represent the existence of a double dip at $h\nu \sim 2.23$ eV and 2.67 eV.

S3. Normalized TAS spectra

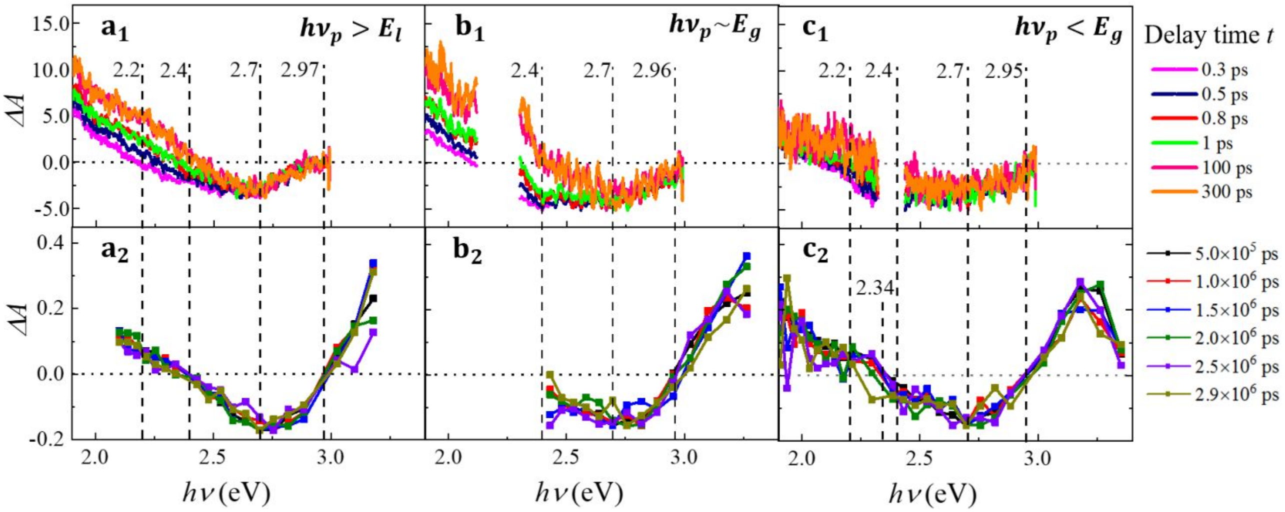

Fig. S3 The normalized TAS spectra (with respect to the absolute dip intensity) under a) $h\nu_p = 3.5\ eV > E_l$, b) $h\nu_p = 2.2\ eV \sim E_g$, and c) $h\nu_p = 1.7\ eV < E_g$. Top row: ps-TAS spectra; Bottom row: ns-TAS spectra.

S4. Reproducibility of TAS spectra

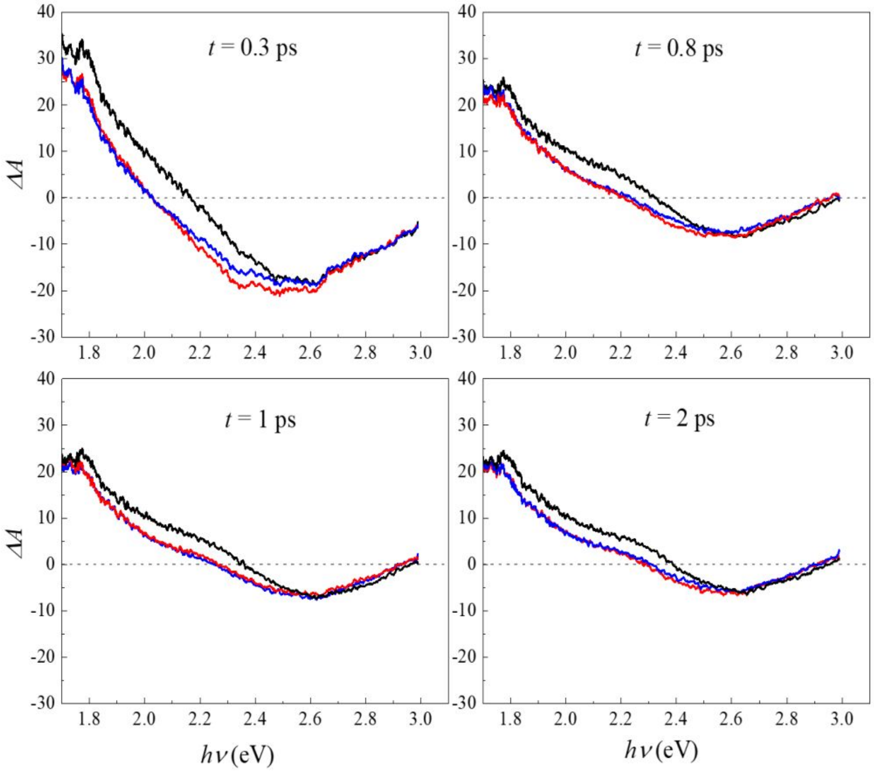

Fig. S4 Time-dependent spectra of three sets of samples under $h\nu_p = 3.5$ eV $> E_l$ (different colors in the plots) prepared over the course of one year.

S5. Examples of time trace analysis

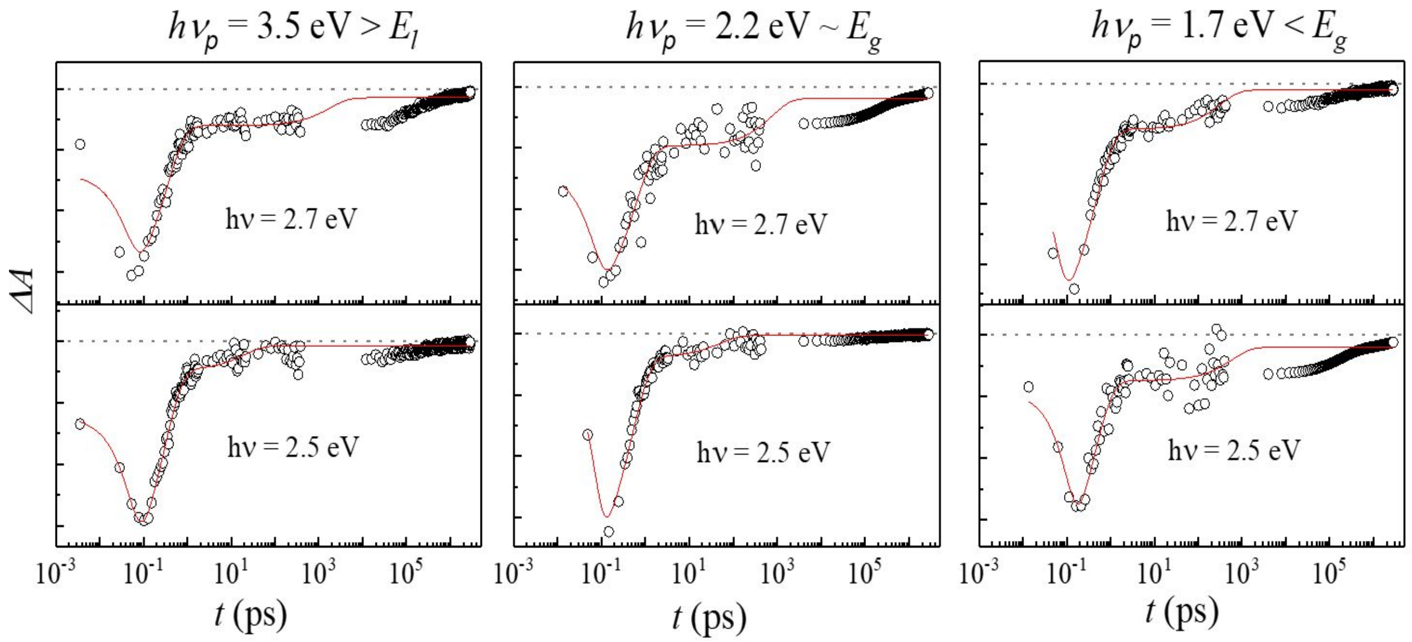

Fig. S5 Examples of exponential fittings of time traces via 2 time constants at $h\upsilon = 2.7$ and 2.5 eV under a) $h\nu_p = 3.5$ eV $> E_l$, b) $h\nu_p = 2.2$ eV$\sim E_g$, and c) $h\nu_p = 1.7$ eV $< E_g$. The red curves indicate fitting results. The horizontal dotted lines indicate $\Delta A = 0$.

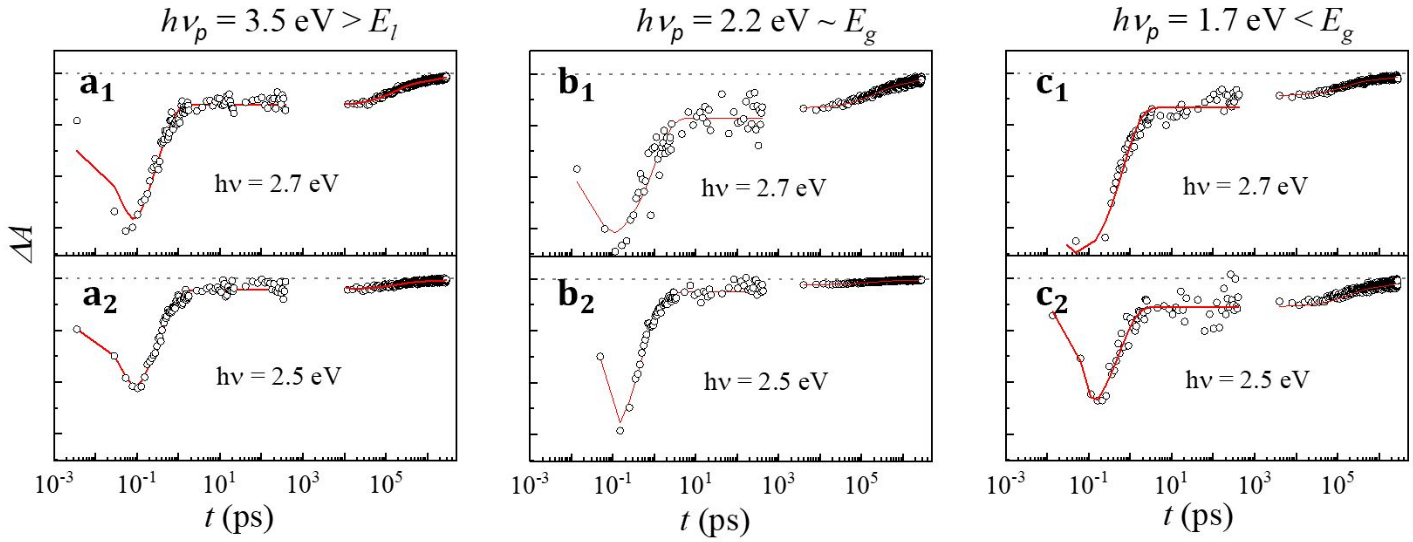

Fig. S6 Examples of exponential fittings of time traces via 3 time constants at $h\upsilon = 2.7$ and 2.5 eV under a) $h\nu_p = 3.5$ eV $> E_l$, b) $h\nu_p = 2.2$ eV$\sim E_g$, and c) $h\nu_p = 1.7$ eV $< E_g$. The red curves indicate fitting results. The horizontal dotted lines indicate $\Delta A = 0$.

S6. Solution of the rate equations

	$h\nu_p > E_l$	$h\nu_p \sim E_g, h\nu_p < E_g$
Δn_v	$-\Delta n \frac{(e^{-k_3 t} k_0 k_1 (k_0 - 3k_1)(k_0 - k_2)(3k_1 - k_2) + e^{-k_2 t} k_0 k_1 (k_0 - 3k_1)(k_0 - k_3)(3k_1 - k_3) - e^{-k_0 t} k_1 (3k_1 - k_2)(3k_1 - k_3)\left(k_0^2 + 3k_2 k_3 - 2k_0(k_2 + k_3)\right) + e^{-3k_1 t} k_0 (k_0 - k_2)(k_0 - k_3)\left(3k_1^2 + k_2 k_3 - 2k_1(k_2 + k_3)\right)}{(k_0 - 3k_1)(k_0 - k_2)(3k_1 - k_2)(k_0 - k_3)(3k_1 - k_3)}$	$-\Delta n \frac{e^{-k_3 t} k_1 (3k_1 - k_2) + e^{-k_2 t} k_1 (3k_1 - k_3) + e^{-3k_1 t}\left(3k_1^2 + k_2 k_3 - 2k_1(k_2 + k_3)\right)}{(3k_1 - k_2)(3k_1 - k_3)}$
Δn_S	$\Delta n \frac{k_0\, k_1 \left((k_0 - 3k_1)e^{-k_2 t} + (3k_1 - k_2)e^{-k_0 t} + (k_2 - k_0)e^{-3k_1 t}\right)}{(k_0 - 3k_1)(k_0 - k_2)(3k_1 - k_2)}$	$\Delta n \frac{k_1\left(e^{-k_2 t} - e^{-3k_1 t}\right)}{3k_1 - k_2}$
Δn_d	$\Delta n \frac{k_0 k_1 \left((k_0 - 3k_1)e^{-k_3 t} + (3k_1 - k_3)e^{-k_0 t} + (k_3 - k_0)e^{-3k_1 t}\right)}{(k_0 - 3k_1)(k_0 - k_3)(3k_1 - k_3)}$	$\Delta n \frac{k_1\left(e^{-k_3 t} - e^{-3k_1 t}\right)}{3k_1 - k_3}$
Δn_{c_1}	$\Delta n k_0 \frac{e^{-3k_1 t} - e^{-k_0 t}}{k_0 - 3k_1}$	$\Delta n e^{-3k_1 t}$
Δn_{c_2}	$\Delta n e^{-k_0 t}$	0

Table S1. Solution of rate equations at the 3 different excitation cases.

S7. Estimation of the initial electron populations in CuO

In the unit cell of CuO crystal, there are two Cu and two O atoms. Considering 2 and 6 valence electrons for Cu and O, respectively, there will be a total of 16 electrons in the unit cell. Since the volume of the unit cell is 44.49 Å^3, the density of the VB electrons $n_{v\infty}$ in equilibrium can be estimated as,

$$n_{v\infty} = \frac{16}{44.486\text{Å}^3} = 3.6 \times 10^{29} m^{-3}. \quad \text{(S1)}$$

$n_{S\infty}$ and $n_{d\infty}$ are estimated based on Boltzmann's distribution at room temperature[1]:

$$n_{s\infty} = n_{v\infty} e^{-\frac{E_s}{k_\beta T}} = 8.7 \times 10^{20} m^{-3}, \quad \text{(S2)}$$

$$n_{d\infty} = n_{v\infty} e^{-\frac{E_d}{k_\beta T}} = 2.09 \times 10^{12} m^{-3}, \quad \text{(S3)}$$

and E_{c_1} and E_{c_2} are assumed to have no population in equilibrium, i.e., $n_{c_1\infty} = n_{c_2\infty} = 0$.

S8. Expressions of $\Delta A(\nu,t)$

According to the argument in the main manuscript, not all the resonant absorptions would contribute to $\Delta A(\nu,t)$ at a particular $h\nu$. Depending on the resonant energies close to $h\nu$ and corresponding spectral widths, only few specific absorption transitions need to be considered. For example, under the $h\nu_p = 1.7\ eV < E_g$ excitation, the explicit expressions for $\Delta A(\nu,t)$ at $h\nu = 2.2$ eV can be written as (4 resonant absorptions are considered),

$$\Delta A(\nu,t) = \Delta A_{dc_2}(\nu,t) + \Delta A_{sc_1}(\nu,t) + \Delta A_{sc_2}(\nu,t) + \Delta A_{vc_1}(\nu,t) = \frac{1}{(3\mathrm{k}_1 - \mathrm{k}_2)(3\mathrm{k}_1 - \mathrm{k}_3)}\Delta\mathrm{n}$$
$$\{e^{-k_3 t}\mathrm{k}_1(3\mathrm{k}_1 - \mathrm{k}_2)\mathrm{N_c}(\mathrm{S}_{\mathrm{dc}_2}(\nu) - \mathrm{S}_{\mathrm{vc}_1}(\nu)) + e^{-k_2 t}\mathrm{k}_1(3\mathrm{k}_1 - \mathrm{k}_3)\mathrm{N_c}(2\mathrm{S}_{\mathrm{sc}_2}(\nu) - \mathrm{S}_{\mathrm{vc}_1}(\nu)) + \Delta\mathrm{n}e^{-(3k_1 + k_3)t}\mathrm{k}_1(3\mathrm{k}_1$$ [illegible]

. (S4)

Since $k_1 \gg k_2 > k_3$ and $\Delta n \ll N_c \sim n_{v\infty}$, Eq. S4 can be simplified as

$$\Delta A(\nu,t) = \frac{1}{3}\Delta\mathrm{n}$$
$$\{e^{-k_3 t}\mathrm{N_c}(\mathrm{S}_{\mathrm{dc}_2}(\nu) - \mathrm{S}_{\mathrm{vc}_1}(\nu)) + e^{-k_2 t}\mathrm{N_c}(2\mathrm{S}_{\mathrm{sc}_2}(\nu) - \mathrm{S}_{\mathrm{vc}_1}(\nu)) - e^{-3k_1 t}[3\mathrm{n}_{\mathrm{v}\infty}\mathrm{S}_{\mathrm{vc}_1}(\nu) + \mathrm{N_c}(\mathrm{S}_{\mathrm{dc}_2}(\nu) + 2\mathrm{S}_{\mathrm{sc}_2}(\nu) + \mathrm{S}$$ [illegible]

(S5)

For $h\nu = 2.7$ eV, the expression for $\Delta A(\nu,t)$ is

$$\Delta A(\nu,t) = \Delta A_{vc_2}(\nu,t) + \Delta A_{Svv}(\nu,t) = \frac{\Delta\mathrm{n}\left(e^{-k_3 t}\mathrm{k}_1(3\mathrm{k}_1 - \mathrm{k}_2) + e^{-k_2 t}\mathrm{k}_1(3\mathrm{k}_1 - \mathrm{k}_3) + e^{-3k_1 t}(3\mathrm{k}_1^2 + \mathrm{k}_2\mathrm{k}_3 - 2\mathrm{k}_1(\mathrm{k}_2 + \mathrm{k}_3))\right)(\mathrm{n_{sv}}\mathrm{S_{svv}}(\nu) - \mathrm{N_c}\mathrm{S}_{\mathrm{vc}_2}(\nu))}{(3\mathrm{k}_1 - \mathrm{k}_2)(3\mathrm{k}_1 - \mathrm{k}_3)}. \quad \text{(S6)}$$

Under $k_1 \gg k_2 > k_3$ and $\Delta n \ll N_c \sim n_{v\infty}$, it becomes

$$\Delta A(\nu,t) = \frac{\Delta\mathrm{n}\left(e^{-k_3 t} + e^{-k_2 t} + e^{-3k_1 t}\right)(\mathrm{n_{sv}}\mathrm{S_{svv}}(\nu) - \mathrm{N_c}\mathrm{S}_{\mathrm{vc}_2}(\nu))}{3}. \quad \text{(S7)}$$

The results show that independent of the spectral region, $\Delta A(\nu,t)$ can be expressed as the summation of terms that each include an exponential decay with only a single decay rate, thus implying that the 3 experimentally resolved time constants, can be directly used in the simulations and they are assumed to correspond to the transitions in **Fig. 4.**

S9. Estimation of the $\sigma_{i\to j}$ and γ_{ij} for each individual absorption transition

The $\sigma_{i\to j}$ and γ_{ij} are estimated using the experimental ps-TAS spectra under $h\nu_p = 3.5$ eV $> E_l$ following the procedure below:

Step 1: Each $\Delta A(\nu,t)$ spectrum at different *t* is fitted by the sum of 7 absorption peaks/dips according to Eq. 9 in the manuscript. Each peak/dip is written as,

$$A_{ij}(t) = \frac{\gamma_{ij}}{2\pi\left[h^2(\nu - \nu_{ij})^2 + \left(\frac{1}{2}\gamma_{ij}\right)^2\right]}\sigma_{i\to j}\left[n_i(t)(N_j - n_j(t)) - n_{i\infty}N_j\right]$$
$$= \frac{\gamma_{ij}}{2\pi\left[h^2(\nu - \nu_{ij})^2 + \left(\frac{1}{2}\gamma_{ij}\right)^2\right]}\eta_{ij}(t), \quad \text{(S8)}$$

where all the time dependent term is defined *via* $\eta_{ij}(t) = \sigma_{i\to j}[n_i(t)(N_j - n_j(t)) - n_{i\infty}N_j]$. During the fitting, ν_{ij} is confined to the following: 2.15 eV $\le E_{c_1} \le$ 2.25 eV, 2.62 eV $\le E_{c_2} \le$ 2.70 eV, 0.45 eV $\le E_s \le$ 0.55 eV, 0.95 eV $\le E_d \le$ 1.05 eV,and -3.25 eV $\le E_{sv} \le -3.15$ eV, and γ_{ij} values are confined to 0.1-0.5 eV. **Figure S7** shows the fitting results.

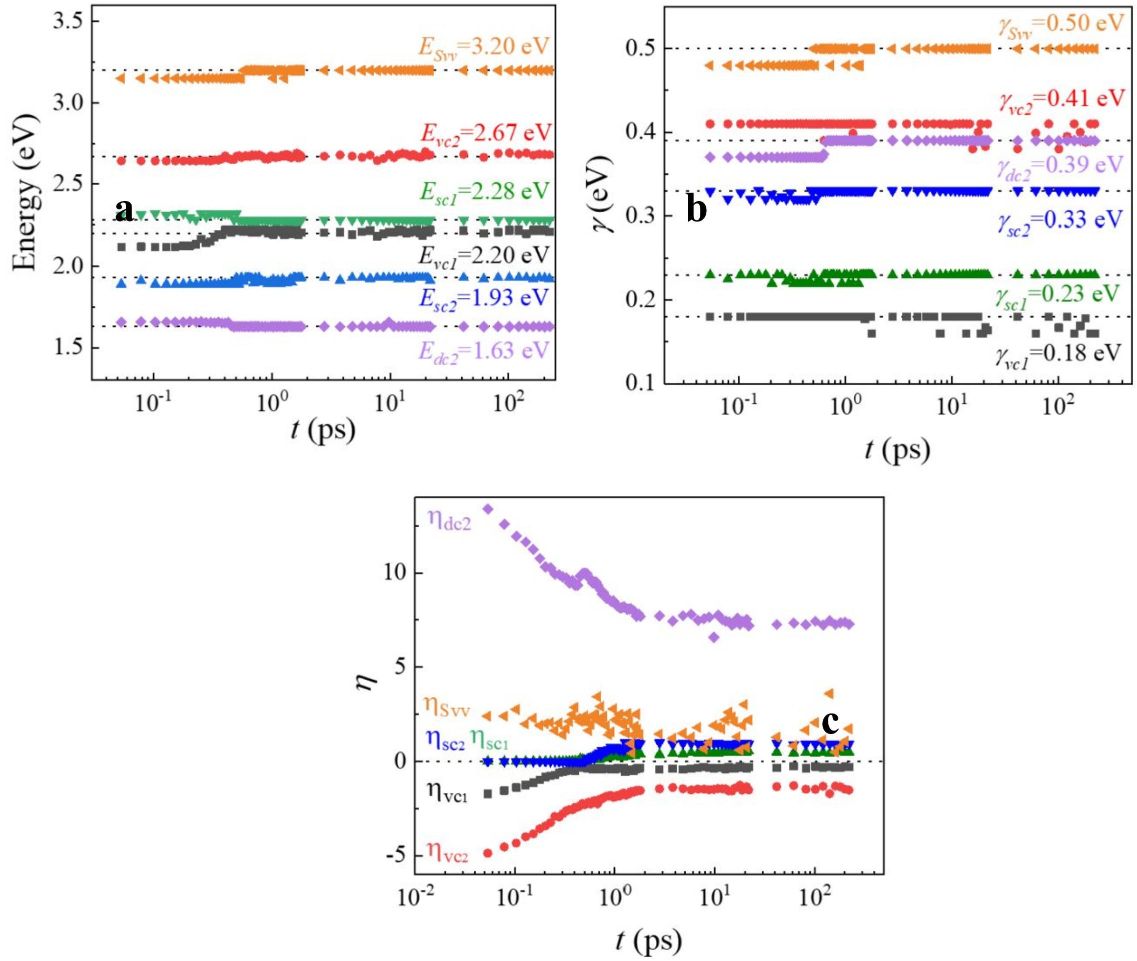

Fig. S7 Estimated model parameters based on the proposed data analysis strategy. (a) energy values of the different energy levels. (b) Lorentzian widths (damping factor), and (c) γ, of the proposed transitions and right: time-dependent η values of the transitions.

Step 2: The fitting result $\eta_{ij}(t)$ (**Fig. S7c**) is further analyzed using the solution $n_i(t)$ of the rate equations and the equilibrium parameters $n_{i\infty} = N_j = 10^{29}\ m^{-3}$ to estimate $\sigma_{i\to j}$. The results are shown in **Fig. S8**.

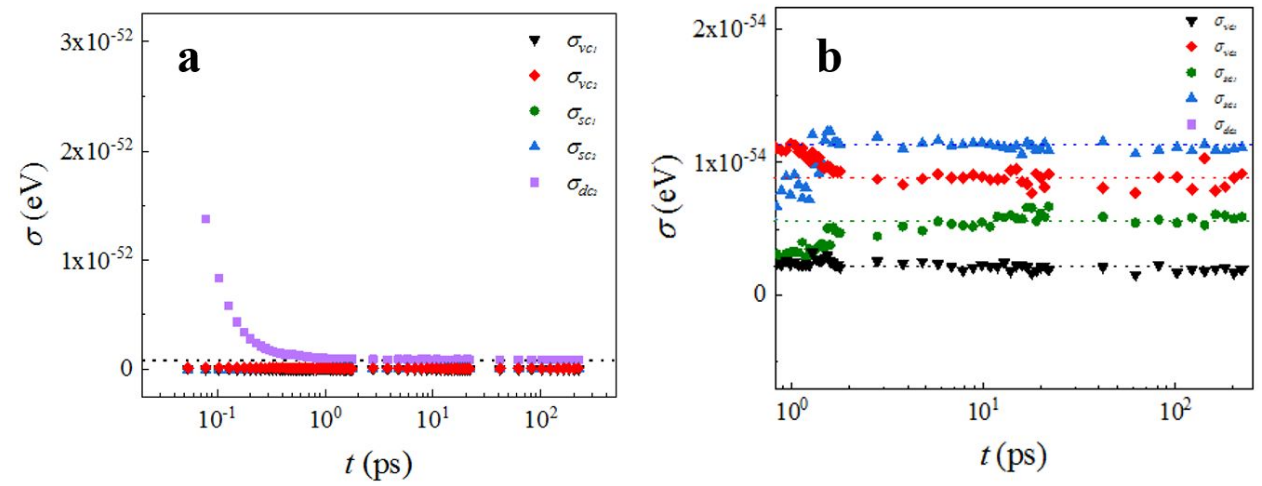

Fig. S8 a) The estimated $\sigma_{i\to j}$ values versus t for the entire ps TAS timescale; b) the zoom-in plot of $\sigma_{i\to j}$ versus t at $t > 1$ ps.

Based on **Fig. S8b**, the estimated values of $\sigma_{i\to j}$ for different transitions are $\sigma_{v\to c_1}$~$2.1 \times 10^{-54} m^7$, $\sigma_{v\to c_2}$~$8.9 \times 10^{-54} m^7$, $\sigma_{s\to c_1}$~$5.9 \times 10^{-54} m^7$, $\sigma_{s\to c_2}$~$1.15 \times 10^{-54} m^7$, $\sigma_{d\to c_2}$~$1.0 \times 10^{-53} m^7$. The estimated values for σ are consistent with the reported values in the literature, considering the correct conversion of the cross-section expressions[2, 3]. The extracted $\sigma_{i\to j}$ values are then used to simulate all the TAS spectra. **Table S2** summarize all the parameters used to simulate the TAS spectra.

Table S2. Parameters to simulate the TAS spectra

$\sigma_{sv\to n_v}$ (m^7)	$\sigma_{n_v\to n_{c_1}}$ (m^7)	$\sigma_{n_v\to n_{c_2}}$ (m^7)	$\sigma_{n_s\to n_{c_1}}$ (m^7)	$\sigma_{n_s\to n_{c_2}}$ (m^7)	$\sigma_{n_d\to n_{c_2}}$ (m^7)
2.1×10^{-54}	2.1×10^{-54}	8.9×10^{-54}	5.9×10^{-54}	1.15×10^{-53}	10^{-53}
$\gamma_{n_{sv}\to n_v}$ (eV)	$\gamma_{n_v\to n_{c_1}}$ (eV)	$\gamma_{n_v\to n_{c_2}}$ (eV)	$\gamma_{n_s\to n_{c_1}}$ (eV)	$\gamma_{n_s\to n_{c_2}}$ (eV)	$\gamma_{n_d\to n_{,c_2}}$ (eV)
0.5	0.18	0.41	0.23	0.33	0.39
E_{sv} (eV)	E_s (eV)	E_d (eV)	E_{c_1} (eV)	E_{c_2} (eV)	
−3.20	0.5	1	2.20	2.67	
k_0 (ps^{-1})	k_1 (ps^{-1})	k_2 (ps^{-1})	k_3 (ps^{-1})		
10	2	5.9×10^{-6}	3.6×10^{-7}		
Δn (m^{-3})					
2.5×10^{24}					

S10. The normalized simulated spectra for three $h\nu_p$ cases

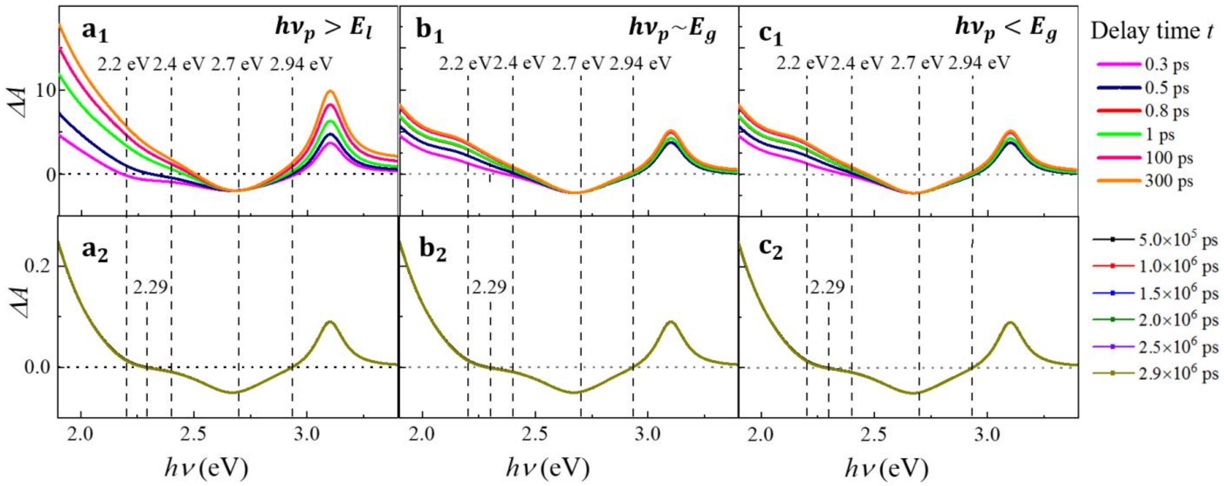

Fig. S9 The normalized simulated TAS spectra $\Delta A(\nu,t)$ under a) $h\nu_p = 3.5\ eV > E_l$, b) $h\nu_p = 2.2\ eV \sim E_g$, and c) $h\nu_p = 1.7\ eV < E_g$. Top row: ps-TAS spectra; Bottom row: ns-TAS spectra.

S8. Description of the videos

M1: The experimental spectral fittings of ps-TAS spectra for the $h\nu_p =$ 3.5 eV case to extract the simulation parameters.

M2: The comparison of the simulated TAS spectra versus the experimental spectra in ps to µs timescale for $h\nu_p =$ 3.5 eV case.

M3: The comparison of the simulated TAS spectra versus the experimental spectra in ps to µs timescale for $h\nu_p =$ 2.2 eV case.

M4: The comparison of the simulated TAS spectra versus the experimental spectra in ps to µs timescale for $h\nu_p =$ 1.7 eV case.

M5: The free electron absorption analysis for $h\nu_p =$ 3.5 eV case.

M6: The free electron absorption analysis for $h\nu_p =$ 2.2 eV case.

M7: The free electron absorption analysis for $h\nu_p =$ 1.7 eV case.

M8: The comparison of the FEA-corrected simulated TAS spectra versus the experimental spectra in ps to µs timescale for $h\nu_p =$ 3.5 eV case.

M9: The comparison of the FEA-corrected simulated TAS spectra versus the experimental spectra in ps to µs timescale for $h\nu_p =$ 2.2 eV case.

M10: The comparison of the FEA-corrected simulated TAS spectra versus the experimental spectra in ps to µs timescale for $h\nu_p =$ 1.7 eV case.

References

1. Lin, Z.; Chen, W.; Zhan, R.; Chen, Y.; Zhang, Z.; Song, X.; She, J.; Deng, S.; Xu, N.; Chen, J., Thermal-Enhanced Field Emission from Cuo Nanowires Due to Defect-Induced Localized States. *AIP Advances* **2015,** *5* (10), 107229.
2. Valenta, J.; Greben, M.; Remeš, Z.; Gutsch, S.; Hiller, D.; Zacharias, M., Determination of Absorption Cross-Section of Si Nanocrystals by Two Independent Methods Based on Either Absorption or Luminescence. *Applied Physics Letters* **2016,** *108* (2), 023102.
3. Lewis, T. R.; Gómez Martín, J. C.; Blitz, M. A.; Cuevas, C. A.; Plane, J. M. C.; Saiz-Lopez, A., Determination of the Absorption Cross Sections of Higher-Order Iodine Oxides at 355 and 532 Nm. *Atmos. Chem. Phys.* **2020,** *20* (18), 10865-10887.
